# Beneficial effects of *Elaeagnus rhamnoides* (L.) A. Nelson and its most abundant flavonoids on the main mechanisms related to diabetic bone disease

**DOI:** 10.1080/13880209.2025.2523392

**Published:** 2025-07-03

**Authors:** Roman Biro, Radoslav Omelka, Anna Sarocka, Noemi Penzes, Veronika Kovacova, Vladimira Mondockova, Monika Martiniakova

**Affiliations:** ^a^ Department of Zoology and Anthropology, Faculty of Natural Sciences and Informatics, Constantine the Philosopher University in Nitra, Nitra, Slovakia; ^b^ Department of Botany and Genetics, Faculty of Natural Sciences and Informatics, Constantine the Philosopher University in Nitra, Nitra, Slovakia

**Keywords:** Sea buckthorn, quercetin, isorhamnetin, kaempferol, diabetic bone disease, hyperglycemia, inflammatory state, insulin resistance, advanced glycation end products

## Abstract

**Context:**

Diabetes mellitus represents a group of metabolic disorders that can adversely affect numerous organ systems, including the skeletal system. It elevates bone fragility and causes secondary osteoporosis, known as diabetic bone disease (DBD). The treatment of DBD depends on the control of hyperglycemia supplemented with anti-osteoporotic agents, but this has unsatisfactory efficiency.

**Objective:**

This article provides a comprehensive review on the effects of *Elaeagnus rhamnoides* (L.) A. Nelson (sea buckthorn; family *Elaeagnaceae*), a prospective anti-diabetic and osteoprotective supplement, and its most abundant flavonoids (quercetin, isorhamnetin, kaempferol) on major mechanisms related to DBD.

**Methods:**

‘Sea buckthorn’ (SB), ‘quercetin’, ‘isorhamnetin’, ‘kaempferol’, ‘DBD’, ‘hyperglycemia’, ‘inflammatory state’, ‘insulin resistance’ (IR), ‘advanced glycation end products’ (AGEs) were used as keywords, and relevant literature was obtained from online databases (PubMed, Web of Science, Scopus).

**Results and Conclusions:**

SB and flavonoids mentioned above exert hypoglycemic and anti-inflammatory properties, attenuate IR, inhibit AGEs formation, thereby positively affecting the main DBD-related mechanisms. The direct effect of SB on DBD has not been investigated yet, but the beneficial impact of quercetin on DBD has been revealed. Therefore, it can be assumed that SB could favorably influence DBD, as its great potential to treat other bone-related diseases (osteoporosis, rheumatoid arthritis) has been reported. Further research, including high-quality *in vitro* and animal model studies, as well as large-scale clinical trials, is needed to confirm such a putative positive effect and to identify more efficient therapies against various diabetic complications, including DBD.

## Introduction

Diabetes mellitus (DM) is a group of metabolic diseases characterized by hyperglycemia resulting from disorders of insulin secretion, insulin action, or both (American Diabetes Association 2010; Blahova et al. 2021). According to Ogurtsova et al. (2022), 536.6 million people live with DM and almost half of them do not know they suffer from this disease. Several types of DM can be identified. The first type is type I diabetes mellitus (T1DM), caused by autoimmune destruction of the pancreatic β cells, leading to absolute insulin deficiency (Kakleas et al. 2015; Asmat et al. 2016). T1DM typically occurs in childhood and early adulthood. Both genetic and environmental factors contribute to the susceptibility to this disease (Kaul et al. 2013). In T1DM, hyperglycemia develops only when ∼90% of β cells are lost (Alam et al. 2014). The second type is type II diabetes mellitus (T2DM), which is considered the most common form of DM. It is characterized by insufficient synthesis of insulin and its secretion, secondary to insulin resistance (IR) (Kaul et al. 2013; Alam et al. 2021). Another type is gestational DM that develops during pregnancy and usually disappears after delivery (Alam et al. 2021).

Many of the most serious complications of DM result from oxidative stress, which is induced by hyperglycemia and hyperlipidemia, important features of this disease. Persistent hyperglycemia directly increases the formation of advanced glycation end products (AGEs), which are associated with inflammation, homeostatic disturbance of the vasculature, and subsequent development of multiple late complications (Moldogazieva et al. 2019; Lee et al. 2022b). In addition to diabetic retinopathy, neuropathy, nephropathy, cardiovascular disease, stroke, diabetic foot, DM can also damage the skeletal system. It causes bone loss and even secondary osteoporosis, also known as diabetic bone disease (DBD) (Costantini and Conte 2019; Martiniakova et al. 2024a). Significantly elevated bone fragility and fracture risk due to reduced bone mass and disturbed bone microarchitecture are its typical manifestations (Ebeling et al. 2022). Moreover, DBD predisposes patients to long-term bone pain and motor dysfunction. The risk of disability and fracture is higher compared to patients with primary osteoporosis (Ferrari et al. 2018). Both T1DM and T2DM have been found to correlate with raising fracture risk. Patients with T1DM are at increased risk of ankle, hip, and total fractures. An elevated risk of humerus, hip, foot, upper leg, and total fractures has been reported in subjects with T2DM (Fan et al. 2016; Moayeri et al. 2017; Wang et al. 2019; Chen et al. 2022; Martiniakova et al. 2024a).

Overall, DBD may be caused by multiple mechanisms such as hyperglycemia, inflammatory state, insulin and insulin-like growth factor-1 (IGF-1) deficiency, accumulation of AGEs, increased levels of sclerostin and leptin, lower levels of bone formation and bone resorption markers (Vigevano et al. 2021; Wu et al. 2022; Cavati et al. 2023; Martiniakova et al. 2024a). Studies investigating the pathogenesis of DBD focus mainly on hyperglycemia, pro-inflammatory cytokine production, IR, and AGEs formation.

In addition to pharmacological treatment of various diabetic complications, natural hypoglycemic molecules derived from plants have recently gained an importance (Omelka et al. 2020; Blahova et al. 2021). Their use has great potential, especially due to fewer side effects. *Elaeagnus rhamnoides* (L.) A. Nelson (sea buckthorn, SB) is a flowering shrub, belonging to the family *Elaeagnaceae* that is naturally widespread in Asia and Europe (Yang and Kallio 2002). It has the ability to survive even under rigorous climatic conditions, tolerating temperatures ranging from −40 °C to +40 °C and high soil pH levels (Ciesarová et al. 2020; Ren et al. 2020). The ripe berries are oval in shape and typically yellow, orange, or red, depending on the variety. Two primary products can be obtained from the berries: juice from the pulp and oil extracted from the seeds (Ciesarová et al. 2020). Various parts of this medicinal plant contain a large amount of bioactive substances which, due to antioxidant, anti-inflammatory, and immunomodulatory activities, play a critical role in modulating oxidative stress and treating inflammatory, infectious diseases (Chagas et al. 2022), as well as aging, metabolic, and degenerative disorders (Abdel-Daim et al. 2018b). Furthermore, compounds with antioxidant activity are valuable in mitigating drug- and chemical-induced toxicity, scavenging reactive oxygen species (ROS), oxidative damage to DNA and lipids, redox homeostasis, or apoptosis (Abdel-Daim et al. 2018a). Nearly 200 physiologically active metabolites have been identified in SB. In general, carotenoids, phenolic metabolites (particularly phenolic acids and flavonoids), vitamins (mainly vitamin C), fatty acids, phytosterols, organic acids, amino acids, and minerals are included among the main bioactive metabolites. Flavonoids, known as phytoconstituents and secondary plant metabolites are considered the most abundant active metabolites in SB (He et al. 2023). Moreover, they have recently been proposed as anti-diabetic by virtue of their hypoglycemic action (Górniak et al. 2019; Mihal et al. 2023), as well as anti-osteoporotic due to their ability to improve disturbed bone microarchitecture and strength (Martiniakova et al. 2024b). Quercetin, isorhamnetin, and kaempferol are generally among the most widespread flavonoids in SB that play a key role in attracting pollinators and seed dispersers (Chagas et al. 2022) and fulfill both aforementioned characteristics.

In general, flavonoids can be extracted from SB (especially from fruits, leaves, and twigs) using a variety of methods, including solvent extraction, ultrasound-, enzyme-, or microwave-assisted extraction. The extraction is usually carried out using 70-85% ethanol or methanol, most often in a material to liquid ratio of 1:10, at various temperatures (from room temperature to 90 °C) and durations (from 2 to 48 h) (Olas et al. 2018; He et al. 2023; Norouzi et al. 2024). Recently, ionic liquids and natural deep eutectic solvents have emerged as alternatives to organic solvents, providing flavonoid yields of around 20 mg/g, which is 1.3 to 2.4 times the yields of traditional extraction methods (Cui et al. 2018). If ultrasound- or microwave-assisted extraction is used, the extraction time, sound frequency, and microwave power fall within 30 s to 30 min, 40 to 2450 kHz, and 25 to 750 W, respectively. The extracted material should be characterized by multiple analytical methods, such as thin-layer chromatography, high-performance or ultra-performance liquid chromatography with mass spectrometry or diode-array detection, gas chromatography with flame ionization detection, or NMR spectroscopy, to determine the composition of the extract (He et al. 2023; Norouzi et al. 2024).

The resulting composition of SB extracts depends on both the source (fruits, leaves, seeds) and the extraction method used. Flavonoids tend to be dominant compounds in the phenolic fraction of SB berries; Olas et al. (2016) determined their total amount to 214.04 mg/g. Identified flavonols typically comprise isorhamnetin, quercetin, and kaempferol present as glycosides and aglycones (Teleszko et al. 2015), especially isorhamnetin-2-Osophoroside-7-O-rhamnoside, isorhamnetin-3-O-rutinoside, isorhamnetin-3-O-glucoside, quercetin-3-O-rutinoside, quercetin-3-O-glucoside (Olas 2016). On the other hand, hydrolysable tannins were dominant constituents of the phenolic fraction of SB leaves and flavonoids were represented by different mono- and diglycosides of isorhamnetin, quercetin and kaempferol, both simple and acylated (Skalski et al. 2018). Total flavonoid content in SB leaves was 74.7 mg/g in the study by Skalski et al. (2019). SB seed extract contained various flavonoids (total flavonoid content was 157.2 mg/g), among which dominant compounds included 3-O-dihexoside-7-O-deoxyhexosides of kaempferol, isorhamnetin, and quercetin, as well as isorhamnetin 3-O-glucoside-7-O-rhamnoside (Sławińska et al. 2023, 2024). Modifications in extraction methods or solvent concentrations yielded various fractions with different composition (Sławińska et al. 2024).

**Figure 1.**
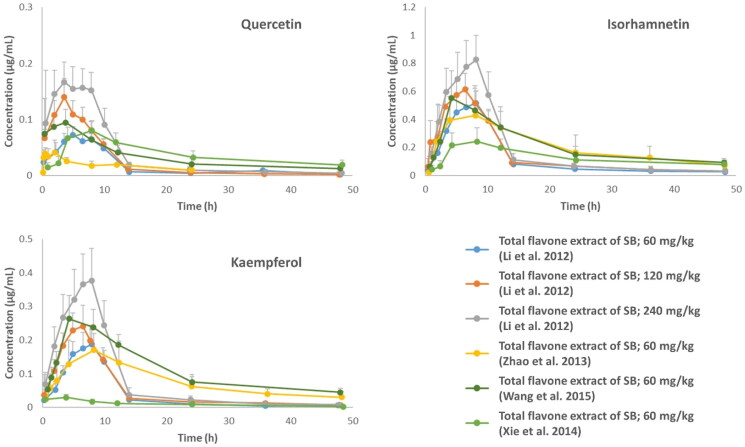
Plasma concentration-time curves of quercetin, isorhamnetin, and kaempferol after oral administration of total flavone extracts of SB at different doses. The curves were constructed based on data presented in the studies by (Li et al. 2012; Zhao et al. 2013; Xie et al. 2014; Wang et al. 2015).

When administered orally, flavonoids from SB are absorbed by both passive diffusion and active transport. Other factors, such as enterohepatic circulation, drug efflux pumps, and intestinal microflora, also participate in the absorption processes (Liu et al. 2021). In pharmacokinetic studies using rats administered by total SB flavones extracts, quercetin was absorbed more rapidly than isorhamnetin and kaempferol (Figure 1, Table 1) (Li et al. 2012; Zhao et al. 2013; Xie et al. 2014; Wang et al. 2015). Furthermore, isorhamnetin, kaempferol, and in some cases quercetin showed no “double-peak” phenomenon in concentration-time curves, suggesting weak enterohepatic recirculation or a potential effect of co-occurring components in SB extracts. On the other hand, all three flavonoids, in pure form or in extracts, exhibited the “double-peak” phenomenon in most human trials (Erlund et al. 2000; DuPont et al. 2004; Schulz et al. 2005; Riva et al. 2019; Solnier et al. 2023). Flavonoids are mainly present as glycosides and are released as aglycones in the digestive tract. The rate of hydrolysis may vary for different compounds, resulting in flavonoid aglycones being released at different times. In addition, flavonoids show a low bioavailability due to their low lipophilicity and poor aqueous solubility. Therefore, various technologies have been used to improve the delivery and solubility of flavonoids, even those directly isolated from SB. These methods include solid dispersion, self-emulsifying formulations, lecithin-based delivery systems, phospholipid complexes, self-microemulsion delivery system, co-administration with phytic acid as an absorption enhancer, or nanosuspensions. In some cases, the methods mentioned above were able to achieve up to 15-20 times higher absorption of flavonoids (Zhao et al. 2013; Xie et al. 2014; Wang et al. 2015; Guo et al. 2017; Riva et al. 2019; Tian et al. 2020; Solnier et al. 2023). Biodegradable polymer microspheres, which can be used as drug carriers and have the potential for bone regeneration, are also gaining importance (Tao et al. 2023; Song et al. 2025). Flavonol glycosides and glucuronides remain in the blood for a relatively long time after consumption of SB, with quercetin being eliminated most rapidly in rats (Table 1). The co-occurring components in SB extracts could significantly slow down the elimination when compared to the pure form (Li et al. 2012).

**Table 1.** Pharmacokinetic parameters of quercetin, isorhamnetin, and kaempferol after application of total flavone extracts of SB (TFSB) and pure metabolites (according to available studies).

Flavonoids	Model	AUC_(0-t)_ (mg h/L)	t_max_ (h)	t_1/2_ (h)	C_max_ (μg/mL)	MRT (h)	Reference
**Quercetin**							
TFSB (60 mg/kg)	rat	0.58 ± 0.15	5.58 ± 2.78	4.95 ± 3.23	0.09 ± 0.24	5.72 ± 1.19	Li et al. (2012)
TFSB (120 mg/kg)	rat	1.05 ± 0.48	4.50 ± 1.82	4.89 ± 2.54	0.14 ± 0.63	5.75 ± 1.01	Li et al. (2012)
TFSB (240 mg/kg)	rat	1.88 ± 1.03	4.25 ± 1.84	4.77 ± 5.12	0.19 ± 0.71	7.31 ± 1.51	Li et al. (2012)
TFSB (60 mg/kg)	rat	0.83 ± 0.40	1.50 ± 0.71		0.04 ± 0.01	19.73 ± 7.35	Zhao et al. (2013)
TFSB (60 mg/kg)	rat	1.92 ± 0.55	2.60 ± 1.34	24.24 ± 4.90	0.10 ± 0.02		Wang et al. (2015)
TFSB (60 mg/kg)	rat	0.49 ± 0.15	1.90 ± 1.92	15.23 ± 2.66	0.03 ± 0.01		Xie et al. (2014)
Quercetin (8 mg)	human	0.630	1.90 ± 1.20	17.10 ± 3.70	0.041		Erlund et al. (2000)
Quercetin (20 mg)	human	0.882	2.70 ± 1.90	17.70 ± 2.60	0.066		Erlund et al. (2000)
Quercetin (50 mg)	human	1.138	4.90 ± 2.10	15.10 ± 3.00	0.086		Erlund et al. (2000)
Quercetin (500 mg)	human	0.08 ± 0.01	3.00 ± 3.20		0.01 ± 0.01		Solnier et al. (2023)
Quercetin (500 mg)	human	0.08 ± 0.02	4.83 ± 0.52	6.26 ± 1.26	0.01 ± 0.00	6.84 ± 0.40	Riva et al. (2019)
**Isorhamnetin**							
TFSB (60 mg/kg)	rat	6.50 ± 2.19	7.08 ± 1.96	51.91 ± 59.67	0.59 ± 0.22	18.73 ± 6.35	Li et al. (2012)
TFSB (120 mg/kg)	rat	8.36 ± 1.47	6.00 ± 1.55	45.31 ± 23.10	0.63 ± 0.24	22.02 ± 6.81	Li et al. (2012)
TFSB (240 mg/kg)	rat	11.25 ± 4.89	7.00 ± 1.82	50.80 ± 50.51	0.85 ± 0.41	20.76 ± 10.88	Li et al. (2012)
TFSB (60 mg/kg)	rat	13.74 ± 4.33	6.40 ± 2.19		0.47 ± 0.16	30.90 ± 3.13	Zhao et al. (2013)
TFSB (60 mg/kg)	rat	14.07 ± 3.83	5.60 ± 2.19	32.54 ± 4.61	0.57 ± 0.19		Wang et al. (2015)
TFSB (60 mg/kg)	rat	8.96 ± 3.58	7.20 ± 1.79	27.67 ± 1.18	0.25 ± 0.10		Xie et al. (2014)
Isorhamnetin (1 mg/kg)	rat	1.62 ± 0.46	7.20 ± 2.30	11.20 ± 2.00	0.07 ± 0.00		Lan et al. (2007)
Isorhamnetin (50 mg/kg)	rat	4.12 ± 0.33		6.73 ± 0.37	0.34 ± 0.29	3.63 ± 0.28	Peng et al. (2017c)
**Kaempferol**							
TFSB (60 mg/kg)	rat	1.72 ± 0.69	7.08 ± 1.96	4.42 ± 2.61	0.22 ± 0.07	8.46 ± 2.52	Li et al. (2012)
TFSB (120 mg/kg)	rat	2.46 ± 1.06	6.00 ± 1.55	10.07 ± 6.66	0.25 ± 0.12	25.79 ± 14.47	Li et al. (2012)
TFSB (240 mg/kg)	rat	4.60 ± 2.02	7.00 ± 1.82	28.09 ± 28.58	0.39 ± 0.18	17.08 ± 8.15	Li et al. (2012)
TFSB (60 mg/kg)	rat	5.24 ± 2.59	6.80 ± 2.68		0.18 ± 0.08	29.33 ± 2.48	Zhao et al. (2013)
TFSB (60 mg/kg)	rat	7.19 ± 1.43	5.60 ± 2.19	28.70 ± 5.58	0.28 ± 0.06		Wang et al. (2015)
TFSB (60 mg/kg)	rat	2.58 ± 0.85	7.20 ± 1.79	28.28 ± 9.61	0.08 ± 0.02		Xie et al. (2014)
Kaempferol (20 mg/kg)	rat	0.23 ± 0.06	1.78 ± 1.75	4.03 ± 2.13	0.04 ± 0.01	5.75 ± 1.66	Li et al. (2022)
Kaempferol (100 mg/kg)	rat	0.76 ± 0.10	1.50 ± 0.80				Barve et al. (2009)
Kaempferol (250 mg/kg)	rat	1.30 ± 0.04	1.00				Barve et al. (2009)

AUC_(0-t)_, area under the plasma concentration-time curve from zero to the time of the last quantifiable concentration; C_max_, maximum plasma concentration; MRT, mean residence time; t_1/2_, terminal half-life; t_max_, time to maximum plasma concentration; TFSB, total flavone extract of SB.

The aim of this review was to summarize the current knowledge from available studies on the effects of SB and its most numerous flavonoids (quercetin, isorhamnetin, and kaempferol) on major mechanisms related to DBD, including hyperglycemia, inflammatory state, IR, and AGEs formation. For each mechanism, its relationship to diabetic bone was indicated. Relevant literature was retrieved from online databases (PubMed, Web of Science, Scopus; 1996-October 2024) and the terms ‘SB’, ‘quercetin’, ‘isorhamnetin’, ‘kaempferol’, ‘DBD’, ‘hyperglycemia’, ‘inflammatory state’, ‘IR’, ‘AGEs’ were used as keywords. Potential selection bias was eliminated by rigorously searching literature sources. The outcomes for the main part of the review included biochemical, morphological, cellular, and molecular parameters related to DBD, hyperglycemia, inflammatory state, IR, and AGEs. Studies were assessed for eligibility in accordance with the PRISMA guidelines (Page et al. 2021). In the individual studies, where it was relevant, we evaluated the fulfillment of the requirements for the phytochemical characterization of the plant extracts according to ConPhyMP (Heinrich et al. 2022). The aforementioned flavonoids were chosen not only because they are the most abundant in SB, but also because they favorably influence all mechanisms related to DBD. Since the direct effect of SB on DBD is still unknown and the positive impact of quercetin on DBD has already been described (as the only flavonoid characterized in this review), we included it in the manuscript in order to make this review clearer and more comprehensive. Synergistic effects between quercetin and kaempferol, kaempferol and epicatechin were also characterized, supporting the hypothesis that SB could alleviate DBD. Overall, this review reveals for the first time the complex relationships between SB and its best-known flavonoids and the key mechanisms associated with DBD.

## The impacts of SB and its most widespread flavonoids on hyperglycemia

Hyperglycemia is the most obvious clinical manifestation of DM, which is often accompanied by hyperlipidemia and increased oxidative stress. It can adversely affect many tissues and cells, including bone cells. Reduced osteoblast differentiation, elevated osteoblast apoptosis, and enhanced osteoclast-mediated bone resorption result from hyperglycemia, leading to impaired bone microstructure and strength (Omelka et al. 2020; Wu et al. 2022).

In a study by Sharma (2011), SB fruit pulp (1 and 2 mL/kg for 3 weeks) was found to reduce blood glucose level in streptozotocin (STZ)-induced diabetic rats. However, this study did not provide important details on the plant material used (Table 2). Zhang et al. (2010) demonstrated the hypoglycemic properties of an aqueous extract of SB seed residues (400 mg/kg for 4 weeks) in STZ-induced diabetic rats. In both studies mentioned above, increased antioxidant status was also noted. According to Dupak et al. (2022), SB berries (500 mg/kg for 3 months) also reduced the onset of hyperglycemia in Zucker diabetic fatty (ZDF) rats. However, used material would require better characterization. In addition to hypoglycemic effects, Table 2 also lists other positive impacts of SB application in relation to diabetic complications.

**Table 2.** The detailed characteristics and main findings of included studies.

**Mechanism related to DBD**	**Applied treatment and metabolite description**	**Research model**	**Main outcomes**	**Reference**
**Hyperglycemia**	sea buckhorn (*Hippophae rhamnoides* L.) (1 and 2 mL/kg for 3 weeks) Herbal parts: fruit pulp Origin: purchased from Panacea International, New Delhi, India Minimal active dose: 1 mL/kg No data about authentication of the plant material, locality and date of harvesting, deposition of voucher specimen, details about plant material processing.	*In vivo*: male albino Wistar rats with STZ- and nicotinamide-induced DM - single intraperitoneal (i.p.) injection of STZ (60 mg/kg), 15 min after the i.p. administration of 120 mg/kg nicotinamide (n = 6 per group) Control: positive and negative (n = 6 per group)	↓ Blood glucose ↑ GSH level ↓ HbA1c ↓ MDA ↓ degenerative changes of pancreatic β cells	Sharma (2011)
	sea buckhorn (400 mg/kg per day for 4 weeks) Herbal parts: seeds Authentication: Hongqing Li Origin: Chifeng, Inner Mongolia Date of harvesting: November 2006 Deposition of voucher specimen: no.: Wang S.Y. 2006001, university herbarium Extraction details: solvent (distilled water), type (liquid), drug to solvent ratio 1:10, drug to extract ratio 100:9.41 Methods for extract characterization: phytochemical screening, phenol–sulfuric acid method and spectrophotometry Minimal active dose: 400 mg/kg	*In vivo*: male Sprague-Dawley rats with STZ-induced DM – i.p. injection of STZ (45 mg/kg) (n = 12 per group) Control: positive and negative (n = 12 per group)	↓ Blood glucose, ↓ Triglyceride ↑ SOD activity ↑ GSH level ↓ NO	Zhang et al. (2010)
	sea buckhorn (500 mg/kg for 3 months) Herbal parts: berries Origin: Institute of Biodiversity of Agriculture in Nitra, Slovak Republic Plant material processing: fruits homogenized in distilled water Minimal active dose: 500 mg/kg Methods for material characterization: HPLC-DAD, LC-MS, GC-FID No data about authentication of the plant material, date of harvesting, deposition of voucher specimen.	*In vivo*: ZDF rats with T2DM (n = 8 per group) Control: positive and negative (n = 8 per group)	↓ Hyperglycemia ↓ Sorbitol levels in the lens	Dupak et al. (2022)
	Quercetin (0.04% or 0.08% of the diet for 6 weeks) Manufacturer and/or supplier of the product: Sigma Chemical Co. (St. Louis, MO, USA) Product name: Quercetin Minimal active dose: 0.04% of the diet	*In vivo*: male C57BL/KsJ-db/db mice (n = 6 per group) Control: negative (n = 6 per group)	↓ Blood glucose ↓ HOMA-IR ↓ Triglycerides ↓ Total cholesterol (0.04%) ↑ HDL-cholesterol (0.04%) ↑ SOD, CAT, GSH activity ↑ Plasma adiponectin (0.04%)	Jeong et al. (2012)
	Quercetin (10 and 15 mg/kg i.p. per day for 10 days) Manufacturer and/or supplier of the product: Sigma Chemical Co. (St. Louis, MO, USA) Product name: Quercetin Minimal active dose: 10 mg/kg i.p.	*In vivo*: adult male Sprague–Dawley rats with STZ-induced DM - single i.p. injection of STZ (40 mg/kg) (n = 5 per group) Control: positive and negative (n = 5 per group)	↓ Blood glucose ↓ Total cholesterol ↓ Triglycerides	Vessal et al. (2003)
	Quercetin (300 mg/kg for 8 weeks) Origin: extracted from *Eucommia ulmoides* Oliv. and Gardenia (purity ≥96% via HPLC) Minimal active dose: 300 mg/kg	*In vivo*: adult Sprague-Dawley rats with high fat diet and STZ- induced DM - single i.p. injection of STZ (35 mg/kg) (n = 8 per group) Control: positive and negative (n = 8 per group)	↓ Blood glucose ↓ HbA1c ↓ Triglycerides ↓ Total cholesterol ↓ LDL-cholesterol ↑ HDL-cholesterol	Lai et al. (2021)
	Quercetin (15 mg/kg per day for 4 weeks) Manufacturer and/or supplier of the product: Sigma Chemical Co. (St. Louis, MO, USA) Product name: Quercetin Minimal active dose: 15 mg/kg	*In vivo*: male Wistar albino rats with STZ-induced DM - single i.p. injection of STZ (50 mg/kg) (n = 10 per group) Control: positive and negative (n = 10 per group)	↓ Blood glucose ↑ Plasma insulin, calcium, and magnesium ↑ BV/TV, Tb.N, Tb.Th ↑ Biomechanical properties	Kanter et al. (2007)
	Quercetin (10 and 50 mg/kg for 12 weeks) Manufacturer and/or supplier of the product: Sigma Chemical Co. (St. Louis, MO, USA) Product name: Quercetin Minimal active dose: 10 mg/kg	*In vivo*: male Sprague-Dawley rats with STZ-induced DM - single i.p. injection of STZ (30 mg/kg) (n = 8 per group) Control: positive and negative (n = 8 per group)	↓ Blood glucose ↓ HbA1c ↓ Total cholesterol ↓ LDL-cholesterol ↓ Triglyceride ↑ Expression and activity of SIRT1 ↑ AKT signaling	Peng et al. (2017a)
	Isorhamnetin (10, 20, and 40 mg/kg for 3 weeks) Manufacturer and/or supplier of the product: Sigma Chemical Co. (St. Louis, MO, USA) Product name: Isorhamnetin Minimal active dose: 10 mg/kg	*In vivo*: male Wistar rats with high fat diet and STZ-induced DM - single i.p. injection of STZ (30 mg/kg) (n = 10 per group) Control: positive and negative (n = 10 per group)	↓ Blood glucose and insulin (40 mg/kg) ↓ HOMA-IR (20 mg/kg) ↓ Total cholesterol (20 mg/kg) ↓ LDL-cholesterol (20 mg/kg) ↑ HDL-cholesterol (20 mg/kg) ↓ Triglycerides (40 mg/kg) ↓ m-TOR, IGF1-R expression (10-20 mg/kg) ↑ AKT2 mRNA, miR-1, and miR-3163 expression (10-20 mg/kg)	Matboli et al. (2021)
	Isorhamnetin (10 mg/kg for 10 days) Manufacturer and/or supplier of the product: Sigma-Aldrich (Hamburg, Germany) Product name: Isorhamnetin Minimal active dose: 10 mg/kg	*In vivo*: male C57BL/6 mice with high fat diet and STZ-induced DM - single i.p. injection of STZ (40 mg/kg) (n = 6 per group) Control: positive and negative (n = 6 per group)	↓ Blood glucose and insulin ↓ HOMA-IR ↓ Triglycerides ↓ Total cholesterol ↓ LDL-cholesterol ↑ GSH ↓ MDA, IL-6 ↑ GLUT4 and AMPK protein expression	Alqudah et al. (2023)
	Kaempferol (50, 100, 200 mg/kg for 45 days) Manufacturer and/or supplier of the product: Sigma Chemical Co. (St. Louis, MO, USA) Product name: Kaempferol Minimal active dose: 50 mg/kg	*In vivo*: male Wistar rats with STZ-induced DM - single i.p. injection of STZ (40 mg/kg) (n = 6 per group) Control: positive and negative (n = 6 per group)	↓ Blood glucose (maximum effect at 100 mg/kg) ↑ Plasma insulin ↓ Lipid peroxidation markers (100 mg/kg) ↑ SOD, CAT, GSH (100 mg/kg)	Al-Numair et al. (2015)
	Kaempferol (5 and 10 mg/kg for 30 days) Origin: extracted from *Eruca sativa* (characterized by NMR and mass spectroscopy). Minimal active dose: 5 mg/kg	*In vivo*: male Wistar rats with STZ-induced DM (n = 6 per group) Control: positive and negative (n = 6 per group)	↓ Serum glucose ↑ Serum insulin ↑ SOD, GSH ↓ AGEs level in sciatic nerve ↓ TNF-α, TGF-β and IL-1β in sciatic nerve	Kishore et al. (2018)
**Inflammatory state**	sea buckhorn oil (100 mg/kg p.o. + 40 μL/paw) Herbal parts: fruit pulp Origin: Food and Herbal Park, Patanjali Ayurveda Limited, Haridwar, India Minimal active dose: 100 mg/kg Characterization of the material: only content of saturated, polyunsaturated, and monounsaturated fat determined by GC–FID No data about authentication, voucher specimen deposit, locality and date of harvesting, details about plant material processing.	*In vivo*: Wistar rats with Carrageenan-induced paw edema (n = 8 per in group) Control: negative	↓ Paw volume and edema	Balkrishna et al. (2019)
	sea buckhorn (1.25-50 µL/mL for 24 h) Herbal parts: fruit pulp Origin: Food and Herbal Park, Patanjali Ayurveda Limited, Haridwar, India Minimal active dose: 1.25 µL/mL Characterization of the material: only content of saturated, polyunsaturated, and monounsaturated fat determined by GC–FID No data about authentication, voucher specimen deposit, locality and date of harvesting, details about plant material processing.	*In vitro*: LPS-stimulated THP-1 cells (National Centre for Cell Science, Pune, India) Control: negative	↓ Cellular reactive nitrogen species (0.3-10 µL/mL) ↓ LPS-stimulated release of IL-1β and IL-6 (1.25-5 µL/mL) ↓ NF-κB expression (0.3-10 µL/mL)	Balkrishna et al. (2019)
	sea buckhorn high-purity flavonoids extract (100 μg/mL for 24 h) Herbal parts: fruit Authentication: X. Chen (Northeast Forestry University, China), botanical identification of species Locality: Linzhi, Tibet Autonomous Region, China Minimal active dose: 100 µg/mL Extraction details: solvent (deep eutectic solvent composed of 1,4-butanediol and choline chloride at a molar ratio of 1:2), type (liquid), drug to solvent ratio 1:30; subsequent purification using macroporous resin Methods for material characterization: HPLC-MS	*In vitro*: LPS-stimulated RAW264.7 cells (Yuchi Biotechnology Co. Ltd., Shanghai, China) Control: positive and negative	↓ expression of IL-6, TNF-α, and IL-1β ↓ nitric oxide production ↓ apoptosis	Yang et al. (2025)
	sea buckhorn leaves (parallel methanol fraction; 0.05, 5, 50 µg/mL for 48 h) Herbal parts: leaves Authentication: ethno-botanical identification of species Voucher specimen is preserved at Defence Institute of High Altitude Research, Leh, India Locality: hilly regions of western Himalayas, India Date of harvesting: September (year not provided) Minimal active dose: 0.05 µg/mL Extraction details: solvent (70% ethanol, hexane, petroleum ether, ethyl acetate, chloroform, 80% ethyl acetate in hexane, methanol), type (liquid), drug to solvent ratio 1:10 Methods for material characterization: HPLC, HPTLC	*In vitro*: peritoneal macrophages from female BALB/c mice Control: negative	↓ LPS-induced NO production ↑ TNF-α, IL-6, IFN-γ ↓ iNOS and COX-2 expressions	Tanwar et al. (2018)
	sea buckhorn (0.5–100 μg/mL for 24 h and 48 h) Herbal parts: leaves Extraction details: solvent (ethanol), type (liquid, supercritical CO₂ technology), drug to extract ratio 100:1.77 Minimal active dose: 50 μg/mL Methods for material characterization: HPLC No data about origin, authentication, voucher specimen deposit, locality and date of harvesting.	*In vitro*: mouse macrophage cell line MH-S (American Type Culture Collection, Manassas, VA) Control: negative	↓ LPS-induced secretion of IL-6, TNF‐α and NO production ↓ Expression of CD40, iNOS and COX-2 proteins ↓ NF-κB activation	Jayashankar et al. (2012)
	sea buckhorn (270 μg/kg for 18 days) Herbal parts: leaves Extraction details: solvent (ethanol), type (liquid, supercritical CO₂ technology), drug to extract ratio 100:1.77 Minimal active dose: 270 μg/mL Methods for material characterization: HPLC No data about origin, authentication, voucher specimen deposit, locality and date of harvesting.	*In vivo*: BALB/c mice with adjuvant-induced arthritis − 100 μL of complete Freund’s adjuvant intradermally into the foot pad (n = 4 per group) Control: positive and negative (n = 4 per group)	↓ Inflammation of paw edema	Jayashankar et al. (2012)
	Quercetin (0.1-80 μM for 30 min) Manufacturer and/or supplier of the product: Sigma Chemical Co. (St. Louis, MO, USA) Product name: Quercetin Minimal active dose: 40 μM	*In vitro*: LPS-stimulated human neutrophils Control: negative	↓ LPS-induced secretion of IL-6, intracellular IL-6 level and IL-6 mRNA expression	Liu et al. (2005)
	Quercetin (3, 30 μM for 30 min) Manufacturer and/or supplier of the product: Sigma-Aldrich (St. Louis, MO, USA) Product name: Quercetin Minimal active dose: 3 μM	*In vitro*: PMACI-stimulated HMC-1 cells Control: positive and negative	↓ TNF-α, IL-1β, IL-6, and IL-8 expression ↓ PMACI-induced activation of NF-kB and p38 MAPK	Min et al. (2007)
	Quercetin (5, 10, 20, and 40 μM for 12, 24 and 48 h) Manufacturer and/or supplier of the product: National Institute for the Control of Pharmaceutical and Biological Products Product name: Quercetin Minimal active dose: 5 μM	*In vitro*: high glucose-induced human mesangial cells (HMC) - high glucose (30 mM) Control: negative	↓ High glucose-induced cell proliferation ↓ % of G1 cells ↓ High glucose-induced NF-κB and MCP-1 expression	Chen et al. (2012)
	Quercetin (2.5, 5, 10 or 20 µM for 1h) Manufacturer and/or supplier of the product: HWI Analytik (Rheinzabern, Germany) Product name: Quercetin Minimal active dose: 2.5 μM	*In vitro*: human retinal pigment epithelial (ARPE-19) cells Control: negative	↓ IL-1β-stimulated expression of ICAM-1, IL-6, IL-8 and MCP-1 ↓ Phosphorylation of MAPKs (ERK, p38, JNK) ↓ IL-1β-stimulated NF-κB activation	Cheng et al. (2019)
	Quercetin (0.1, 0.5, 1, 5 and 10 µM for 4h) Manufacturer and/or supplier of the product: Sigma Chemical Co. (Madrid, Spain) Product name: Quercetin Minimal active dose: 0.1 μM	*In vitro*: human hepatoma cell line HepG2 Control: negative	↓ TNF-α-induced activation of NF-κB ↓ TNF-α-induced ROS production ↓ TNF-α-induced COX-2 levels	Granado-Serrano et al. (2012)
	Quercetin (25, 50 and 100 µM for 24h) Manufacturer and/or supplier of the product: Sigma-Aldrich (St. Louis, MO, USA) Product name: Quercetin Minimal active dose: 25 μM	*In vitro*: LPS-stimulated human peripheral blood mononuclear cells (PBMCs) Control: negative	↑ LPS-inhibited viability of PBMCs ↓ LPS-induced secretion of TNF-α, IL-1β, and IL-6 ↓ LPS-increased levels of NF-κB	Zhang et al. (2016)
	Quercetin (30 µg/mL for 24h) Manufacturer and/or supplier of the product: National Institute for the Control of Pharmaceutical and Biological Products (Beijing, China) Product name: Quercetin Minimal active dose: 30 μg/mL	*In vitro*: TNF-α-induced human umbilical vein endothelial cells (HUVECs) Control: negative	↓ TNF-α induced apoptosis ↓ Upregulation of VCAM-1, ICAM-1 ↓ Activation of NF-κB and AP-1	Chen et al. (2020)
	Quercetin (50 mg/kg per day for 6 weeks) Manufacturer and/or supplier of the product: Xi’an App-Chem Bio, (Xi’an, China) Product name: Quercetin Minimal active dose: 50 mg/kg	*In vivo*: albino rats with STZ-induced DM - single i.p. injection of STZ (50 mg/kg) (n = 8 per group) Control: positive and negative (n = 8 per group)	↓ TNF-α and CRP levels ↓ NF-κB activation ↓ HOMA-IR ↓ Systolic blood pressure ↓ Leukocyte infiltration in the adventitia, endothelial cells pyknosis, and collagen deposition within aorta sections	Mahmoud et al. (2013)
	Quercetin (25, 50 and 100 mg/kg for 6 days) Manufacturer and/or supplier of the product: Aladdin reagent Co. Ltd. (China) Product name: Quercetin Minimal active dose: 25 mg/kg	*In vitro*: BALB/c Kunming mice with LPS/d-GalN-induced liver injury - i.p. injection of LPS (50 μg/kg)/d-GalN (300 mg/kg) (n = 10 per group) Control: positive and negative (n = 10 per group)	↓ LPS/d-GalN-induced expression of TNF-α, IL-1β, and IL-6 ↓ LPS/d-GalN-induced activation of NF-κB ↓ Phosphorylation of MAPKs (ERK, p38, JNK) ↓ BAX, BCL-2, CASP3, CASP8, and CASP9 expression	Peng et al. (2017b)
	Quercetin (50 mg/kg per day for 3 days) Manufacturer and/or supplier of the product: Sigma-Aldrich (St. Louis, MO, USA) Product name: Quercetin Minimal active dose: 50 mg/kg	*In vivo*: BALB/c mice with LPS-induced myocardial dysfunctions (n = 5 per group) Control: positive and negative (n = 5 per group)	↓ LPS-induced IL-1β, TNF-α, NO levels ↓ Phosphorylation of I-κBα ↓ CASP3/7 activation	Wei et al. (2018)
	Isorhamnetin (10 mg/kg for 10 days) Manufacturer and/or supplier of the product: Sigma-Aldrich (Hamburg, Germany) Product name: Isorhamnetin Minimal active dose: 10 mg/kg	*In vivo*: male C57BL/6 mice with high fat diet and STZ-induced DM - single i.p. injection of STZ (40 mg/kg) (n = 6 per group) Control: positive and negative (n = 6 per group)	↓ IL-6 levels	Alqudah et al. (2023)
	Isorhamnetin (50 and 150 mg/kg for 12 weeks) Manufacturer and/or supplier of the product: Sigma-Aldrich (St. Louis, MO, USA) Product name: Isorhamnetin Minimal active dose: 50 mg/kg	*In vivo*: Sprague-Dawley rats with high-fat diet and STZ-induced DM - single i.p. injection of STZ (30 mg/kg) (n = 10 per group) Control: negative (n = 10 per group)	↓ NF-κB and phosphorylated NF-κB ↓ TNF-α, IL-1β, IL-6, ICAM-1, and TGF-β1 (protein and mRNA)	Qiu et al. (2016)
	Kaempferol (12.5 and 25 μg/mL for 2 h) Manufacturer and/or supplier of the product: National Institutes for Food and Drug Control (Beijing, China) Product name: Kaempferol Minimal active dose: 12.5 μg/mL	*In vitro*: LPS plus ATP-induced primary cardiac fibroblasts (rats) Control: negative	↓ TNF-α, IL-1β, IL-6, and IL-18 production ↓ LPS plus ATP-induced NF-κB p65 activation ↓ LPS plus ATP-induced AKT phosphorylation	Tang et al. (2015)
	Kaempferol (0.02, 0.2 and 2 µg/mL for 6 days) Manufacturer and/or supplier of the product: Sigma-Aldrich (St. Louis, MO, USA) Product name: Kaempferol Minimal active dose: 0.02 μg/mL	*In vitro*: THP-1 human monocyte cells Control: negative	↓ IL-32-induced monocyte-macrophage differentiation ↓ IL-32-induced production of TSLP, IL-1β, TNF-α, and IL-8 ↓ IL-32-induced activation of p38, NF-κB, and CASP1	Nam et al. (2017)
	Kaempferol (1 and 5 μM for 1 h) Manufacturer and/or supplier of the product: Shaanxi Huike Botanical Development Cooperation (Beijing, China) Product name: Kaempferol Minimal active dose: 1 μM	*In vitro*: rat prostate endothelial cell line YPEN-1 Control: negative	↓ AGE-induced generation of reactive species ↓ AGE-induced NF-κB activation by suppressing NADPH oxidase	Kim et al. (2010)
	Kaempferol (2 and 4 mg/kg for 10 days) Manufacturer and/or supplier of the product: Shaanxi Huike Botanical Development Cooperation (Beijing, China) Product name: Kaempferol Minimal active dose: 2 mg/kg	*In vivo*: Fischer 344 rats aged 24 months Control: negative	↓ AGE and RAGE expression ↓ NF-κB DNA-binding activity ↓ MMP-9, MCP-1, VCAM-1, and ICAM-1 expression	Kim et al. (2010)
	Kaempferol (25, 50 and 100 mg/kg for 21 days) Manufacturer and/or supplier of the product: Sigma-Aldrich Chemie Gmbh, (Steinheim, Germany) Product name: Kaempferol Minimal active dose: 25 mg/kg	*In vivo*: Swiss mice with STZ-induced DM - single i.p. injection of STZ (200 mg/kg) (n = 8 per group) Control: positive and negative (n = 8 per group)	↓ Blood glucose ↓ Serum IL-1β, TNF-α, GSH, MDA, NO	Abo-Salem (2014)
	Kaempferol (30 and 150 mg/kg for 10 weeks) Manufacturer and/or supplier of the product: ZeLang Medicine Science and Technology Co., Ltd. (Nanjing, China) Product name: Kaempferol Minimal active dose: 30 mg/kg	*In vivo*: New Zealand White rabbits fed high-cholesterol diet (n = 6 per group) Control: positive and negative (n = 6 per group)	↓ serum TNF-α, IL-1β, MDA ↑ serum SOD activity ↓ ICAM-1, VCAM-1 and MCP-1 gene and protein expression in aorta	Kong et al. (2013)
**Insulin resistance**	sea buckhorn (1.8% of sea buckthorn leaves extract – SL; 0.04% of flavonoid glycosides extract from sea buckthorn leaves - SLG; for 12 weeks) Herbal parts: dried leaves Extraction details: solvent (80% ethanol; 20%, 30%, and 50% aqueous ethanol), type (liquid), drug to extract ratio 100:37.4 Methods for extract characterization: HPLC, NMR Minimal active dose: 1.8% and 0.04% w/w No data about origin, authentication of the plant material, locality and date of harvesting, deposition of voucher specimen.	*In vivo*: C57BL/6J mice with high fed diet-induced obesity (n = 10 per group) Control: positive and negative (n = 10 per group)	↓ Blood glucose (SL, SLG) ↓ Triglycerides (SL, SLG) ↓ Total cholesterol (SL) ↓ HDL-cholesterol (SL) ↓ HOMA-IR (SL, SLG) ↓ Insulin and leptin (SL, SLG) ↓ Plasma TNF-α, IL-1β, IL-6 (SL, SLG)	Kwon et al. (2017)
	sea buckthorn flavonoids (purity > 65%, content: quercetin 6.47 ± 0.34 mg/g, isorhamnetin 4.56 ± 0.42 mg/g, catechin 3.83 ± 0.31 mg/g) (0.06% and 0.31% w/w for 14 weeks) Origin: purchased from Shanghai Yuan Ye Biotechnology, Ltd. (Shanghai, China) Methods for material characterization: HPLC Minimal active dose: 0.06% w/w No data about herbal parts, authentication of the plant material, locality and date of harvesting, deposition of voucher specimen, details about plant material processing.	*In vivo*: male C57BL/6J mice with high-fat and high-fructose diet-induced obesity (n = 15 per group) Control: positive and negative (n = 15 per group)	↓ Blood glucose and insulin ↓ HOMA-IR ↓ Triglycerides ↓ Total cholesterol ↓ LDL-cholesterol ↑ HDL-cholesterol ↓ NF-κB activation ↓ COX-2 and IL-1β expression in the brain	Mulati et al. (2020)
	sea buckthorn fruit oil (50, 100, 200, and 400 μM for 24 h) Herbal parts: fruit Origin: purchased from Ordos Conseco Ecological Development Co. (Ordos, China) Methods for material characterization: gas chromatography Minimal active dose: 200 μM No data about authentication of the plant material, locality and date of harvesting, deposition of voucher specimen, details about plant material processing.	*In vitro*: HepG2 cells treated with insulin Control: positive and negative	↑ PI3K expression ↑ Phosphorylation of AKT ↓ GSK-3β expression	Gao et al. (2017)
	sea buckthorn fruit oil (100, 200, and 300 mg/kg per day for 4 weeks) Herbal parts: fruit Origin: purchased from Ordos Conseco Ecological Development Co. (Ordos, China) Methods for material characterization: gas chromatography Minimal active dose: 100 mg/kg No data about authentication of the plant material, locality and date of harvesting, deposition of voucher specimen, details about plant material processing.	*In vivo*: male Sprague-Dawley rats with high-fat diet and STZ-induced DM - single i.p. injection of STZ (30 mg/kg) (n = 9 per group) Control: positive and negative (n = 9 per group)	↓ Blood glucose ↓ Insulin sensitivity	Gao et al. (2017)
	Quercetin (50, 100 mg/kg for 45 days) Manufacturer and/or supplier of the product: Sigma Chemical Co. (St Louis, MO, USA) Product name: Quercetin Minimal active dose: 50 mg/kg	*In vivo*: male Sprague Dawley rats with STZ-induced DM - single i.p. injection of STZ (45 mg/kg) (n = 6 per group) Control: positive and negative (n = 6 per group)	↓ Blood glucose ↓ HbA1c ↑ Insulin secretion ↓ Total cholesterol ↓ LDL-cholesterol ↑ HDL-cholesterol ↑ SOD, CAT ↓ MDA	Arya et al. (2014)
	Quercetin (50 mg/kg per day for 6 weeks) Manufacturer and/or supplier of the product: Xi’an App-Chem Bio (Xi’an, China) Product name: Quercetin Minimal active dose: 50 mg/kg	*In vivo*: albino rats with STZ-induced DM - single i.p. injection of STZ (50 mg/kg) (n = 8 per group) Control: positive and negative (n = 8 per group)	↓ TNF-α and CRP levels ↓ NF-κB activation ↓ HOMA-IR ↓ Systolic blood pressure ↓ Leukocyte infiltration in the adventitia, endothelial cells pyknosis, and collagen deposition within aorta sections	Mahmoud et al. (2013)
	Quercetin (2 and 10 mg/kg daily for 10 weeks) Manufacturer and/or supplier of the product: Sigma Chemicals (Madrid, Spain). Product name: Quercetin Minimal active concentration: 2 mg/kg	*In vivo*: male obese Zucker rats (n = 5 or 7 per group) Control: positive and negative (n = 5 or 7 per group)	↓ HOMA-IR ↓ Triglycerides ↓ Total cholesterol ↓ Insulin ↓ Plasma nitrate plus nitrite (10 mg/kg) ↑ Plasma adiponectin (10 mg/kg) ↓ Systolic blood pressure	Rivera et al. (2008)
	Quercetin (0.04% and 0.08% of the diet for 6 weeks) Manufacturer and/or supplier of the product: Sigma Chemical Co. (St Louis, MO, USA) Product name: Quercetin Minimal active dose: 0.04% of the diet	*In vivo*: male C57BL/KsJ-db/db mice (n = 6 per group) Control: negative (n = 6 per group)	↓ Blood glucose ↓ HOMA-IR ↓ Triglycerides ↓ Total cholesterol (0.04%) ↑ HDL-cholesterol (0.04%) ↑ SOD, CAT, GSH activity ↑ Plasma adiponectin (0.04%)	Jeong et al. (2012)
	Quercetin (10, 30, or 60 µM for 1, 3, 8, and 24 h) Manufacturer and/or supplier of the product: Sigma Chemical Co. (St Louis, MO) Product name: Quercetin Minimal active dose: 10 µM	*In vitro*: human adipocytes treated with TNF-α Control: negative	↓ phosphorylation of Ser307-IRS-1 ↓ PTP-1B gene expression ↑ Insulin stimulated 2-DOG uptake ↓ expression of *IL-6*, *IL-1β*, *IL-8*, and *MCP-*1 genes ↓ IL-6, IL-8, MCP-1 secretion	Chuang et al. (2010)
	Isorhamnetin (10, 20 and 40 mg/kg for 3 weeks) Manufacturer and/or supplier of the product: Sigma Chemical Co. (St Louis, MO, USA) Product name: Isorhamnetin Minimal active dose: 10 mg/kg	*In vivo*: male Wistar rats with high fat diet and STZ- induced DM - single i.p. injection of STZ (30 mg/kg) (n = 10 per group) Control: positive and negative (n = 10 per group)	↓ Blood glucose and insulin (40 mg/kg) ↓ HOMA-IR (20 mg/kg) ↓ Total cholesterol (20 mg/kg) ↓ LDL-cholesterol (20 mg/kg) ↑ HDL-cholesterol (20 mg/kg) ↓ Triglycerides (40 mg/kg) ↓ m-TOR, IGF1-R expression (10-20 mg/kg) ↑ AKT2 mRNA, miR-1, and miR-3163 expression (10-20 mg/kg)	Matboli et al. (2021)
	Isorhamnetin (10 mg/kg for 10 days) Manufacturer and/or supplier of the product: Sigma-Aldrich (Hamburg, Germany) Product name: Isorhamnetin Minimal active dose: 10 mg/kg	*In vivo*: male C57BL/6 mice with high fat diet and STZ- induced DM - single i.p. injection of STZ (40 mg/kg) (n = 6 per group) Control: positive and negative (n = 6 per group)	↓ Blood glucose and insulin ↓ HOMA-IR ↓ Triglycerides ↓ Total cholesterol ↓ LDL-cholesterol ↑ GSH ↓ MDA, IL-6 ↑ GLUT4 and AMPK protein expression	Alqudah et al. (2023)
	Kaempferol (50 mg/kg per day for 6 weeks) Manufacturer and/or supplier of the product: not provided Minimal active dose: 50 mg/kg	*In vivo*: male C57BLKS db/db mice (n = 8 per group) Control group: positive and negative (n = 8 and 7 per group)	↑ Glucose and insulin tolerance ↓ NLRP3, CASP1, and IL-1β protein expression in adipose tissue	Zhai et al. (2024))
	Kaempferol (50, 100, 200 mg/kg for 45 days) Manufacturer and/or supplier of the product: Sigma Chemical Co. (St. Louis, MO, USA) Product name: Kaempferol Minimal active dose: 50 mg/kg	*In vivo*: male Wistar rats with STZ-induced DM - single i.p. injection of STZ (40 mg/kg) (n = 6 per group) Control: positive and negative (n = 6 per group)	↓ Blood glucose (maximum effect at 100 mg/kg) ↑ Plasma insulin (50 mg/kg) ↓ Lipid peroxidation markers (100 mg/kg) ↑ SOD, CAT, GSH (100 mg/kg)	Al-Numair et al. (2015)
	Kaempferol (50 and 150 mg/kg for 10 weeks) Manufacturer and/or supplier of the product: ZeLang Medicine Science and Technology Co., Ltd. (Nanjing, China) Product name: Kaempferol Minimal active dose: 50 mg/kg	*In vivo*: male Sprague-Dawley rats with high fat diet and STZ-induced DM - single i.p. injection of STZ (30 mg/kg) (n = 10 per group) Control: positive and negative (n = 10 per group)	↓ Blood glucose (150 mg/kg) ↓ Serum insulin (50 mg/kg) ↓ HOMA-IR (50 mg/kg) ↓ Triglycerides (50 mg/kg) ↓ Total cholesterol (150 mg/kg) ↓ LDL-cholesterol (50 mg/kg) ↓ IKKβ, NF-κB ↓ TNF-α and IL-6 levels	Luo et al. (2015)
	Kaempferol (1.5 and 2.5 µg/mL for 24 h) Manufacturer and/or supplier of the product: Sigma (St. Louis, MO, USA) Product name: Kaempferol Minimal active dose: 1.5 µg/mL	*In vitro*: HepG2 cells Control: positive and negative	↓ Endoplasmic reticulum stress in thapsigargin-stimulated cells ↓ NF-κB phosphorylation (2.5 µg/mL) ↓ Phosphorylation of IRS-1	Kim et al. (2016)
	Kaempferol (0.1, 1.0, 10 µM for 24 h in *in vitro*) (50 mg/kg for 30, 60, 120 min in *in vivo*) Manufacturer and/or supplier of the product: Tauto Biotech Co., Ltd. (Shanghai, China) Product name: Kaempferol Minimal active dose: 10 µM, 50 mg/kg	*In vitro*: primary human skeletal muscle cells Control: negative *In vivo*: male diet-induced obese C57BL/6 mice (n = 9 per group) Control: negative (n = 9 per group)	↑ Glucose uptake ↑ Akt phosphorylation ↑ AMPK phosphorylation	Moore et al. (2023)
**AGEs**	sea buckthorn leaf and berry extracts (10, 25, 50, 100 μg/mL) Herbal parts: leaves and berries Origin: purchased from Anna Garden (Hoengseong, Gang won-do, Korea) Minimal ‘active dose’ 10 μg/mL Extraction details: solvent (70% ethanol), type (liquid), drug to solvent ratio 1:10, drug to extract ratios 100:28.29 (leaf) and 100:36.02 (berry) Methods for extract characterization: UPLC-MRM-MS for isorhamnetin 3-sophoroside-7-rhamnoside quantification; total phenolic content determined by spectrophotometry No data about authentication of the plant material, locality and date of harvesting, deposition of voucher specimen.	Chemical study not using biological models	↓ AGEs formation (higher in the leaf extract) ↓ AGEs-induced crosslinking to collagen (higher in the leaf extract)	Lee et al. (2022)
	sea buckthorn seed residues (25, 50 and 100 μg/mL for 2-48 h) Herbal parts: seed residues Origin: purchased from Yuhangren Hi-Tech Industrial Co., Ltd. (Hohhot, Inner Mongolia, China) Authentication: Hongqing Li, School of Life Science, East China Normal University (Shanghai, China) Locality and date of harvesting: Chifeng, Inner Mongolia in November 2006 Deposition of voucher specimen: in the University herbarium Minimal active dose: 25 µg/mL Extraction details: solvent (water, petroleum ether, 70% ethanol), type (liquid) Methods for extract characterization: flavonoid content determined by spectrophotometry; total flavonoid content was 69.92 ± 1.84%	*In vitro*: bovine aortic endothelial cells Control: negative and positive (AGEs)	↑ AGEs-injured cell viability ↑ Cellular zinc levels ↓ ROS levels ↑ NO levels ↑ Mn-SOD and NOS activity ↑ Total anti-oxidation competence	Zhuang et al. (2012)
	Quercetin (0.25, 0.5, 1.5, 2.5 mM) Manufacturer and/or supplier of the product: Nanjing Guangrun Biological Products Co., Ltd (Nanjing, Jiangsu, China) Product name: Quercetin Minimal ‘active dose’: 0.25 mM	Chemical study not using biological models	↓ AGEs formation ↑ Trapping reactive dicarbonyl compounds	Li et al. (2014)
	Quercetin (10, 20, 50, 100, 200 and 500 μM) Manufacturer and/or supplier of the product: Sigma Chemical Co. (St. Louis, MO, USA) Product name: Quercetin Minimal active dose: 10 μM	Chemical assays, including those using fresh human blood Control: negative	↓ AGEs formation	Ashraf et al. (2015)
	Quercetin (5, 10, 20, 40, 80 μM for 24 and 48 h) Manufacturer and/or supplier of the product: Sigma Chemical Co. (St. Louis, MO, USA) Product name: Quercetin Minimal active dose: 5 μM	*In vitro*: human gingival fibroblasts Control: negative and positive (AGEs)	↓ AGEs-induced ROS ↓ AGEs-induced cell senescence ↓ IL-6 and IL-8 levels ↓ Activation of NF-κB	Huang et al. (2024)
	Isorhamnetin (20 µM for 10 days) Manufacturer and/or supplier of the product: Sigma-Aldrich (Tokyo, Japan) Product name: Kaempferol Minimal active dose: 20 µM	*In vitro*: human amniotic epithelial stem cells (hAESCs) Control: negative	↓ Gene expression of *COL1A1*, *COL1A2*, *COL4A6*, *FN1*, *MMP-2*, and *SERPINE1*	Kalai et al. (2022)
	Kaempferol (5 and 10 mg/kg for 30 days) Origin: extracted from *Eruca sativa* (characterized by NMR and mass spectroscopy). Minimal active dose: 5 mg/kg	*In vivo*: male Wistar rats with STZ-induced DM (n = 6 per group) Control: positive and negative (n = 6 per group)	↓ Serum glucose ↑ Serum insulin ↑ SOD, GSH ↓ AGEs level in sciatic nerve ↓ TNF-α, TGF-β and IL-1β in sciatic nerve	Kishore et al. (2018)
	Kaempferol (2 and 4 mg/kg for 10 days) Manufacturer and/or supplier of the product: Shaanxi Huike Botanical Development Cooperation (Beijing, China) Product name: Kaempferol Minimal active dose: 2 mg/kg	*In vivo*: Fischer 344 rats aged 24 months Control: negative	↓ AGE and RAGE expression ↓ NF-κB DNA-binding activity ↓ MMP-9, MCP-1, VCAM-1, and ICAM-1 expression	Kim et al. (2010)
	Kaempferol (20 mg/kg for 28 days) Manufactured and/or supplier of the product: Sigma Chemical Co. (St. Louis, MO) Product name: Kaempferol Minimal active dose: 20 mg/kg	*In vivo*: male Wistar rats with STZ-induced DM - single i.p. injection of STZ (70 mg/kg) (n = 12 per group) Control: negative (n = 12 per group)	↓ Blood glucose ↓ Serum levels of AGEs ↑ SOD, GSH, CAT ↓ MDA ↓ TNF-α and IL-6 ↓ MAPK/AGE-RAGE activation	Suchal et al. (2017)

Abbreviations: ↑ increase, ↓ decrease; AGEs, advanced glycation end products; AKT, protein kinase B; AMPK, AMP-activated protein kinase; AP-1, activating protein 1; ATP, adenosine triphosphate; BAX, BCL2-associated X protein; BCL-2, apoptosis regulator; BV/TV, relative bone volume; CASP, caspase; CAT, catalase; COL1A1, collagen type I alpha 1 chain; COL1A2, collagen type I alpha 2 chain; COL4A6, collagen type IV alpha 6 chain; COX-2, cyclooxygenase-2; CRP, C-reactive protein; ERK, extracellular signal-regulated kinase; DM, diabetes mellitus; FN1, fibronectin 1; GC-FID, gas chromatography with flame ionization detection; GLUT4, glucose transporter 4; GSH, glutathione peroxidase; GSK-3β, glycogen synthesis kinase 3beta; HbA1c, glycosylated hemoglobin; HDL, high-density lipoprotein; HOMA-IR, homeostasis model assessment for insulin resistance; HPLC-DAD, high-performance liquid chromatography with diode-array detection; HPLC-MS, high-performance liquid chromatography with mass spectrometry; HPTLC, high-performance thin layer chromatography; ICAM-1, intercellular adhesion molecule 1; IFN-γ, interferon gamma; IGF1-R, insulin-like growth factor 1 receptor; I-κBα, inhibitor of NF-κB; IKKβ, IκB kinase beta; IL, interleukin; iNOS, inducible nitric oxide synthase; i.p., intraperitoneal; IRS-1, insulin receptor substrate 1; JNK, C-jun N-terminal kinase; LC-MS, liquid chromatography mass spectrometry; LDL, low-density lipoprotein; LPS, lipopolysaccharide; LPS/d-GaIN, D-galactosamine/lipopolysaccharide; MAPK, mitogen-activated protein kinase; MCP-1, monocyte chemoattractant protein-1; MDA, malondialdehyde; miR, micro RNA; MMP-2, matrix metalloproteinase 2; MMP-9, matrix metalloproteinase 9; mTOR, mammalian target of rapamycin; NADPH, nicotinamide adenine dinucleotide phosphate; NF-κB, nuclear factor kappa B; NLRP3, NLR family pyrin domain containing 3; NMR, nuclear magnetic resonance spectroscopy; NO, nitric oxide; NOS, nitric oxide synthase; PBMCs, peripheral blood mononuclear cells; PI3K, phosphatidylinositol-3-kinase; PMACI, phorbol 12-myristate 13-acetate and calcium ionophore A23187; PTP-1B, protein tyrosine phosphatase 1B; RAGE, receptor for AGE; ROS, reactive oxygen species; SERPINE1, serpin family E member 1; SIRT1, sirtuin 1; SOD, superoxide dismutase; STZ, streptozotocin; Tb.N, trabecular number; Tb.Th, trabecular thickness; TGF-β, transforming growth factor beta; TNF-α, tumor necrosis factor alpha; TSLP, thymic stromal lymphopoietin; UPLC-MRM-MS, ultra performance liquid chromatography with multiple reaction monitoring mass spectrometry; VCAM-1, vascular cell adhesion protein 1; ZDF, zucker diabetic fatty.

Considering flavonoids in SB, Jeong et al. (2012) and Vessal et al. (2003) reported that quercetin (0.04% or 0.08% of the diet for 6 weeks; 10 and 15 mg/kg i.p. for 10 days, respectively) ameliorated hyperglycemia, dyslipidemia, and improved antioxidant status in diabetic mice and rats. The findings of Lai et al. (2021), Kanter et al. (2007), and Peng et al. (2017c) also supported the fact that quercetin (300 mg/kg for 8 weeks, 15 mg/kg for 4 weeks, 10 and 50 mg/kg for 12 weeks, respectively) is able to reduce hyperglycemia and hyperlipidemia in STZ-induced diabetic rats.

Fasting blood glucose levels showed lower values in diabetic mice treated with isorhamnetin (10 mg/kg for 10 days), as well as in diabetic rats after isorhamnetin administrations (40 mg/kg for 3 weeks) (Matboli et al. 2021; Alqudah et al. 2023).

**Figure 2.**
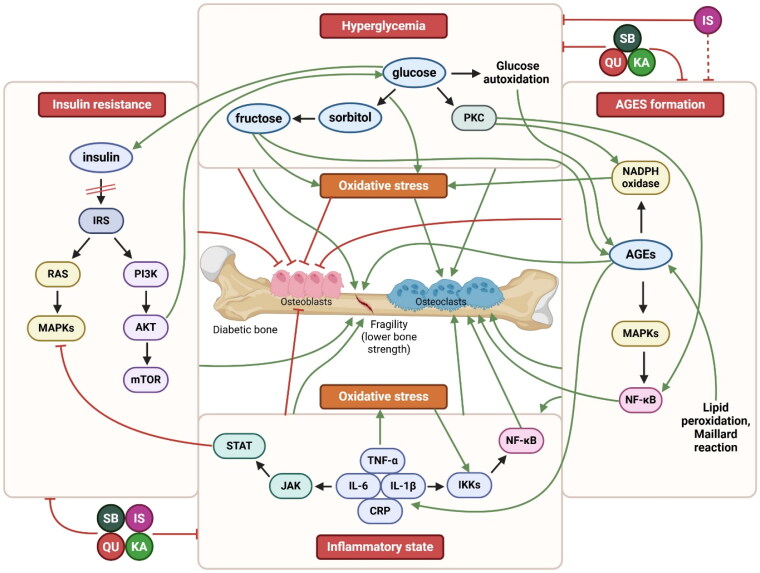
Effects of SB and its most widespread flavonoids on major mechanisms associated with diabetic bone (according to available *in vitro* and *in vivo* studies). The major mechanisms associated with diabetes and diabetic bone disease include AGEs formation, hyperglycemia, insulin resistance, and inflammatory state. AGEs are formed as a result of the Maillard reaction, a process that is accelerated in hyperglycemic conditions. Additionally, AGEs can be produced through glucose autoxidation, the polyol pathway, and lipid peroxidation. AGEs trigger various signaling pathways, including MAPKs and NF-κB, as well as NADPH oxidase, leading to increased oxidative stress and inflammation. The accumulation of AGEs in bone is negatively correlated with the material and biomechanical properties of both cortical and trabecular bone. Higher levels of AGEs are also associated with the production of inflammatory cytokines and oxidative stress, resulting in inflammation and bone resorption. Furthermore, AGEs contribute to decreased bone formation and mineralization. Hyperglycemia and its associated hyperosmolarity have detrimental effects on osteoblast maturation, bone formation, and calcium uptake. Hyperglycemia-induced acidosis may also exacerbate bone resorption, ultimately increasing the risk of fractures. Insulin resistance can be caused by defects at the insulin receptor or post-receptor level, compromising signal transduction, or by an initial alteration in insulin signaling leading to insulin hypersecretion. Insulin signaling, originating from IRS, can be either metabolic or mitogenic. Metabolic signaling is mediated through PI3K and AKT, affecting various substrates such as mTOR, which regulates protein synthesis. Mitogenic signaling targets RAS, activating a cascade that leads to MAPKs, thereby mediating cell growth, division, migration, and apoptosis. Insulin resistance is associated with decreased bone strength and reduced bone turnover. In osteoblasts, insulin resistance lowers the levels of osteocalcin. Elevated levels of inflammatory cytokines lead to oxidative stress production and inhibit osteoblast differentiation. Additionally, these cytokines activate NF-κB signaling and promote osteoclast formation and function. SB and its flavonoids exert hypoglycemic and anti-inflammatory properties, attenuate insulin resistance, and inhibit AGEs formation. Blunt red arrows indicate an inhibitory effect, sharp green arrows designate a stimulatory effect. The red dashed arrow shows the effect based only on gene expression related to AGEs signaling and computational analysis. Black arrows indicate the connection of individual mechanisms. AGEs – advanced glycation end products; AKT: protein kinase B; CRP: C-reactive protein; IKKs: IκB kinases; IL-1β: interleukin 1β; IL-6: interleukin 6; IRS – insulin receptor substrate; IS – isorhamnetin; JAK – Janus kinase; KA – kaempferol; MAPKs: mitogen-activated protein kinases; mTOR – mammalian target of rapamycin; NADPH – nicotinamide adenine dinucleotide phosphate; NF-κB: nuclear factor kappa-B; PI3K: phosphoinositide 3-kinase; PKC – protein kinase C; QU – quercetin; SB – sea buckthorn; STAT: signal transducer and activator of transcription. Created with BioRender.com.

According to Al-Numair et al. (2015), kaempferol supplementation (100 mg/kg for 45 days) significantly improved hyperglycemia and antioxidant status in STZ-induced diabetic rats. Kishore et al. (2018) also demonstrated favorable effects of kaempferol (5 and 10 mg/kg for 30 days) on hyperglycemia and pain response in diabetic rats along with modulation of oxidative stress. Figure 2 illustrates the impacts of SB and its most numerous flavonoids on hyperglycemia, as well as other mechanisms leading to DBD.

## The impacts of SB and its most widespread flavonoids on inflammatory state

**Figure 3.**
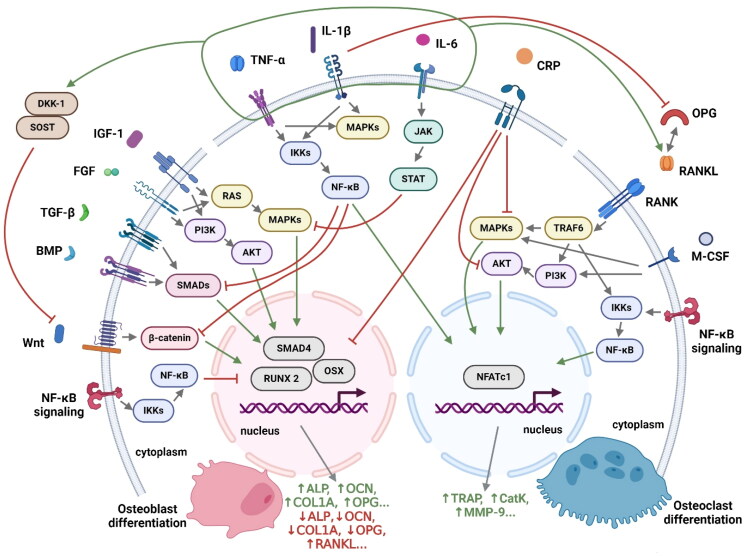
Relationships between pro-inflammatory cytokines and bone signaling pathways affecting osteoblastogenesis and osteoclastogenesis. Several signaling pathways mediate osteoblast differentiation, with the TGF-β, BMP, FGF, IGF-1, Wnt, and NF-κB pathways being among the most important (left). TGF-β and BMP signaling regulate osteoblast-specific gene expression through multiple SMAD proteins. FGF and IGF-1 can regulate osteoblast differentiation via downstream pathways such as PI3K-AKT and MAPK. Wnt binds to receptors, causing β-catenin to accumulate and translocate to the nucleus, where it causes transcription of target genes. Osteoblast differentiation is inhibited by NF-κB signaling with inhibition of SMAD protein activity. The signaling pathways ultimately lead to the regulation of important transcription factors such as RUNX 2, OSX and others, which are essential for the production of specific proteins and osteoblast differentiation. M-CSF, RANKL-RANK, and NF-κB are important molecules and signaling pathways in osteoclastogenesis (right). M-CSF-induced activation results in enhanced osteoclast precursor proliferation and survival through the MAPK and PI3K-AKT pathways. Binding of RANKL to RANK leads to the recruitment of TRAF6 proteins that transduce the signal to downstream targets such as NF-κB or MAPKs. RANKL also activates the PI3K-AKT pathway through TRAF6. In addition, RANKL signaling induces NFATc1, a major transcription factor for osteoclast terminal differentiation, which regulates several osteoclast-specific genes, including TRAP or CatK. Pro-inflammatory cytokines such as TNF-α, IL-1β, IL-6, and CRP are involved in the pathogenesis of chronic inflammation, a key feature of DM and associated complications (top). TNF-α and IL-1β act through several pathways including the activation of NF-κB and MAPKs. IL-6 signals via JAK–STAT pathway. CRP can interact with cell surface receptors and initiate various intracellular signaling cascades. The pro-inflammatory cytokines mentioned above interfere with signaling pathways of osteoblasts and osteoclasts, thereby influencing their development and functions. Additionally, they also up-regulate DKK-1 and SOST, which inhibit Wnt signaling pathway. IL-1β is able to inhibit OPG production. Flavonols, such as quercetin, isorhamnetin, and kaempferol, are able to inhibit the expression and levels of pro-inflammatory cytokines and reduce their negative impact. Blunt red arrows indicate an inhibitory effect, sharp green arrows designate a stimulatory effect. The most common signal transduction is indicated by gray arrows. Green arrows upwards mean increased - stimulated production of molecules, red arrows downwards mean reduced - inhibited production of molecules. AKT: protein kinase B; ALP: alkaline phosphatase; BMP: bone morphogenetic protein; CatK: cathepsin K; COL1A: collagen type I; CRP: C-reactive protein; DKK-1: dickkopf-related protein 1; FGF: fibroblast growth factor; IKKs: IκB kinases; IL-1β: interleukin 1β; IL-6: interleukin 6; JAK: janus kinase; M-CSF: macrophage colony-stimulating factor; MAPKs: mitogen-activated protein kinases; MMP-9: matrix metalloproteinase 9; NF-κB: nuclear factor kappa-B; NFATc1: nuclear factor of activated T-cells 1; OCN: osteocalcin; OPG: osteoprotegerin; OSX: osterix; PI3K: phosphoinositide 3-kinase; RANK: receptor activator of nuclear factor kappa β; RANKL: receptor activator of nuclear factor kappa β ligand; RUNX 2: RUNX family transcription factor 2; SOST: sclerostin; STAT: signal transducer and activator of transcription; TGF-β: transforming growth factor-β; TRAF6: TNF receptor-associated factor 6; TRAP: tartrate-resistant acid phosphatase. Created with BioRender.com.

Chronic inflammatory state in DM is associated with higher levels of pro-inflammatory cytokines such as tumor necrosis factor (TNF)-α, interleukin (IL)-1β, IL-6, or C-reactive protein (CRP) (Rohm et al. 2022). These molecules stimulate oxidative stress and ROS production (Mohamad et al. 2020), resulting in reduced osteoblast differentiation, induced osteoclast differentiation and activity, and increased expression of receptor activator for nuclear factor κB (NF-κB) ligand (RANKL) (Iantomasi et al. 2023). Some of them (e.g., TNF-α) can activate various intracellular signaling molecules, such as Jun NH2-terminal kinase (JNK) and IκB kinase β (IKKβ), leading to impaired insulin action and translocation of NF-κB, an important modulator of osteoclast differentiation (Rohm et al. 2022). However, the effect of pro-inflammatory cytokines on bone health is complex and interferes with signaling pathways affecting osteoblastogenesis and osteoclastogenesis (Figure 3). As a result, pro-inflammatory cytokines promote bone resorption by increasing osteoclast differentiation and activity and/or inhibiting osteoblast differentiation.

Flavonoids exhibit anti-inflammatory properties through various mechanisms, such as inhibition of regulatory enzymes and transcription factors that play a substantial role in controlling mediators involved in inflammation. Moreover, flavonoids are potent antioxidants capable of scavenging ROS and reducing their production. They also modulate immune cells and immune mechanisms that are important in inflammatory processes (Maleki et al. 2019; Martiniakova et al. 2025).

High-purity SB fruit flavonoids extract (100 μg/mL) had significant anti-inflammatory effects, suppressed the expression of nitric oxide synthase, TNF-α, IL-1β, IL-6, and inhibited apoptosis in lipopolysaccharide (LPS)-induced RAW264.7 cells (Yang et al. 2025). The anti-inflammatory effect of SB fruit pulp oil has been identified using LPS-stimulated human monocyte (THP-1) cells and a paw edema rat model. SB oil lowered the production of cellular reactive nitrogen species, reduced the LPS-stimulated release of IL-1β and IL-6 (at SB concentrations of 1.25-5 µl/mL), and inhibited NF-κB protein expression (at SB concentrations of 0.3-10 µl/mL). *In vivo* treatment with SB oil (100 mg/kg; 40 µl/paw) showed a decrease in paw volume and edema (Balkrishna et al. 2019). In this study; however, a more detailed characterization of the plant material used would be desirable. In peritoneal macrophages isolated from BALB/c mice, the methanol fraction of SB leaf extract decreased TNF-α, IFN-γ, and IL-6 levels (Tanwar et al. 2018). Finally, ethanolic extract of SB leaves (100 μg/mL) inhibited LPS-induced secretion of IL-6 and TNF‐α, phosphorylation of p38 mitogen-activated protein kinase (MAPK), and NF-κB p65 translocation in a mouse alveolar macrophage cell line. The *in vivo* mouse model of adjuvant-induced arthritis also showed a significant reduction in the inflammation of paw edema (Jayashankar et al. 2012). However, insufficient details on the material used are a limitation of this study (Table 2).

Many studies have reported that quercetin (0.1-80 µM) can inhibit the expression of TNF-α, IL-6, IL-8, intercellular adhesion molecule 1 (ICAM-1), and monocyte chemoattractant protein-1 (MCP-1) induced by different stimuli in various cell types (Liu et al. 2005; Min et al. 2007; Chen et al. 2012; Cheng et al. 2019). Quercetin has the ability to reduce inflammation through regulation of MAPK and NF-κB signaling pathways *in vitro* (Granado-Serrano et al. 2012; Zhang et al. 2016; Cheng et al. 2019; Chen et al. 2020). In a study by Mahmoud et al. (2013), quercetin (50 mg/kg for 6 weeks) reduced levels of TNF-α and CRP, and prevented the activation of NF-κB in insulin deficient and insulin resistant rat models. Furthermore, experiments in other animal models revealed its ability (50 mg/kg for 3 days, and 25, 50, 100 mg/kg for 6 days) to suppress the production of TNF-α, IL-6, IL-1β and to inhibit the activation of NF-κB and MAPK signaling pathways (Peng et al. 2017a; Wei et al. 2018).

Isorhamnetin (10 mg/kg for 10 days) significantly reduced IL-6 levels in diabetic mice, which was accompanied by antioxidant activities (Alqudah et al. 2023). Similar to quercetin, isorhamnetin (50 and 150 mg/kg for 12 weeks) was able to inhibit the NF-κB signaling pathway (Qiu et al. 2016).

Kaempferol (at doses of 0.7-87 µM) was found to decrease the release of IL-6, IL-18, IL-1β, and TNF-α in LPS plus adenosine triphosphate (ATP)-induced cardiac fibroblasts, or in IL-32-induced human monocyte cell line THP-1. All these effects were accompanied by inhibition of NF-κB (Tang et al. 2015; Nam et al. 2017). Kim et al. (2010) reported that the mechanism to prevent NF-κB activation involves suppression of AGE-induced nicotinamide adenine dinucleotide phosphate (NADPH) oxidase. In the study mentioned above, kaempferol (2 and 4 mg/kg for 10 days) exerted anti-oxidative and anti-inflammatory impacts by modulating the NF-κB signaling cascade and its pro-inflammatory genes (e.g., MMP-9, MCP-1, ICAM-1) in a rat model. The levels of IL-1β and TNF-α were reduced by kaempferol supplementation (25, 50, 100 mg/kg for 21 days) also in diabetic mice (Abo-Salem 2014). In addition, kaempferol (150 mg/kg and 30 mg/kg for 10 weeks) lowered levels of IL-1β, TNF-α, CAM-1 and MCP-1 expression in high cholesterol fed rabbits (Kong et al. 2013).

According to the aforementioned studies, SB flavonoids are able to reduce inflammation *via* several pathways. However, biochemical effects of flavonoids may depend on the molecular structure. Flavonoids exerting both anti-inflammatory and anti-diabetic properties typically have a C2–C3 double bond (C-ring) and hydroxyl groups at the C3’, C4’, C5, and C7 positions of both rings A and B of their skeleton (Shamsudin et al. 2022). Quercetin exactly has this structure; other flavonoids differ slightly at the C3’ position. In addition, it has been demonstrated that substitution at the C3 position of a C-ring decreases the anti-inflammatory action of flavonoids while enhancing their anti-diabetic activity (Shamsudin et al. 2022).

## The impacts of SB and its most widespread flavonoids on IR

Insulin signaling is a conserved pathway that plays a key role in regulating metabolism and longevity, and bone is an insulin-responsive organ. In T2DM, impaired insulin signaling in peripheral tissues results in IR (Tencerova et al. 2019; Guo et al. 2021). Recent studies indicate that T1DM patients also develop IR (Wolosowicz et al. 2020). It is widely recognized that low and high IGF-I levels are related to IR (Friedrich et al. 2012). Significantly lower levels of IGF-1 were determined in patients with DM, leading to reduced bone formation and a higher risk of fragility fractures (Rubin and Patsch 2016; Wu et al. 2022).

Kwon et al. (2017) found that flavonoid glycosides extract from SB leaves (0.04% w/w) improved insulin sensitivity in obese C57BL/6J mice by ameliorating inflammation and decreasing gluconeogenesis. Similarly, Mulati et al. (2020) reported that obese C57BL/6J mice treated with SB flavonoids (0.06% and 0.31% w/w for 14 weeks) showed reduced IR and inflammation. According to Gao et al. (2017), SB fruit oil extract (50, 100, 200, and 400 μM) alleviated T2DM by activating the PI3K/Akt signaling pathway in IR HepG2 cells and improved insulin indices in diabetic rats. However, the plant extracts used in the aforementioned studies would require better characterization (Table 2).

Arya et al. (2014) revealed that quercetin supplementation (50 and 100 mg/kg for 45 days) significantly ameliorated not only IR, but also hyperglycemia and hyperlipidemia in STZ-induced diabetic rats. In addition, oral administration of quercetin (50 mg/kg for 6 weeks) reduced IR also in fructose-induced rat model (Mahmoud et al. 2013). The study by Rivera et al. (2008) indicated that chronic administration of quercetin (2 and 10 mg/kg for 10 weeks) reduced IR, dyslipidemia, and hypertension in the Zucker obese rat model. According to Jeong et al. (2012), homeostasis model assessment of IR (HOMA-IR) showed lower values in diabetic (db/db) mice fed a diet containing 0.04% or 0.08% quercetin for 6 weeks with no significant effect on insulin levels. *In vitro* study by Chuang et al. (2010) showed that quercetin attenuated IR and TNF-α-mediated inflammation in primary human adipocytes. However, IR was determined by measuring the serine phosphorylation of insulin receptor substrate-1 (Ser307-IRS-1) and protein tyrosine phosphatase-1B (PTP-1B) gene expression, and a decrease in insulin-stimulated 2-[3H]deoxyglucose (2-DOG) uptake.

The beneficial effect of isorhamnetin (10 mg/kg for 10 days and 20 mg/kg for 3 weeks) on IR was also determined in diabetic mice and STZ-induced diabetic rats, respectively (Matboli et al. 2021; Alqudah et al. 2023). It was associated with improved glucose and lipid profiles.

Zhai et al. (2024) revealed that kaempferol administration (50 mg/kg for 6 weeks) effectively ameliorated IR in leptin receptor-deficient obese (db/db) mice, an IR model. According to Al-Numair et al. (2015), kaempferol may avoid glucose absorption or improve glucose tolerance by stimulation of insulin secretion. In their study, kaempferol supplementation (50 mg/kg for 45 days) elevated insulin level in STZ-induced diabetic rats. Luo et al. (2015) reported that kaempferol treatment (50 and 150 mg/kg for 10 weeks) may enhance insulin sensitivity in diabetic rats, and possible mechanisms may involve inhibition of IKKβ/NF-κB signaling. The findings of Kim et al. (2016) indicate that kaempferol (1.5 and 2.5 μg/mL for 24 h) can protect against endoplasmic reticulum stress response and hepatic IR (measured by the phosphorylation of IRS-1) in HepG2 cells. According to Moore et al. (2023), kaempferol (10 μmol/L for 24 h) is able to stimulate glucose uptake in skeletal muscle through an AMPK/Akt-dependent mechanism, and therefore may be a viable therapeutic agent for IR.

## Inhibitory effects of SB and its most widespread flavonoids on AGEs formation

AGEs are produced by non-enzymatic glycation and oxidation of proteins, lipids, and nucleic acids. Their effect is exerted *via* interaction with the receptor for AGEs (RAGE) (Oei et al. 2015; Suchal et al. 2017). In general, AGE/RAGE signaling is an intricate cascade that activates multiple intracellular signaling pathways involving NADPH oxidase, MAPKs, and protein kinase C, leading to NF-κB-induced expression of TNF-α, IL-1, IL-6, vascular endothelial growth factor (VEGF), and vascular cell adhesion molecule 1 (VCAM-1) (Kalai et al. 2022). AGEs are incorporated into bone through the non-enzymatic glycation of collagen, resulting in reduced elasticity and increased bone fragility (Rubin and Patsch 2016; Wu et al. 2022). One well-known AGE product is pentosidine, the accumulation of which negatively influence bone strength. According to Oei et al. (2015), T2DM patients with increased pentosidine levels suffered from vertebral fractures. Additionally, urinary pentosidine was associated with a 42% increase in clinical fracture incidence in T2DM (Rubin and Patsch 2016).

Lee et al. (2022a) revealed that SB leaf extract (containing isorhamnetin-3-O-sophoroside-7-O-rhamnoside in a higher concentration) inhibited the formation and crosslinking of AGEs, suggesting that it may have promising impacts against diseases related to AGEs, including DM. However, these findings were obtained using chemical assays only. Moreover, the plant material used was not characterized in detail. *In vitro* study by Zhuang et al. (2012) showed that SB seed residue extracts had favorable effects on AGEs-injured endothelial cells through a nitric oxide-related mechanism, possibly *via* increased cellular zinc levels or its superior antioxidant capacity (Table 2).

According to Li et al. (2014), quercetin effectively inhibited AGEs formation in a dose-dependent manner through scavenging of reactive dicarbonyl metabolites (glyoxal, methylglyoxal), crucial intermediates to form AGEs. Ashraf et al. (2015) also reported that quercetin and its derivatives are able to suppress AGEs accumulation. Moreover, inhibition of glycation formation showed that quercetin was a better and more potent antiglycation agent than aminoguanidine at all stages of glycation. In a study by Huang et al. (2024), quercetin reversed the phosphorylation of p65 and Iκβ in AGEs-stimulated human gingival fibroblasts, indicating that it can modulate the NF-κβ signaling pathway.

Kalai et al. (2022) demonstrated that isorhamnetin significantly reduced gene expression related to AGE/RAGE signaling, such as *COL1A1*, *COL1A2*, *MMP2*, *FN1*, and *SERPINE1*. However, these results were obtained by a stem cell-based tool using human amniotic epithelial cells with whole-genome microarray analysis.

Kishore et al. (2018) revealed that STZ-induced diabetic rats treated with kaempferol (5 and 10 mg/kg for 30 days) showed reduced AGEs formation. Similarly, Kim et al. (2010) demonstrated that kaempferol modulated both AGEs accumulation and RAGE expression in aged rats. Moreover, kaempferol down-regulated AGEs-induced NF-κB signaling. According to Suchal et al. (2017), kaempferol (20 mg/kg for 28 days) has the ability to reduce oxidative stress and AGE-RAGE/MAPK-induced inflammation to attenuate myocardial ischemia/reperfusion injury in diabetic rats.

In addition to positive effects of flavonoids on reducing AGEs and related bone damage through collagen glycation, flavonoids may also be involved in maintaining the integrity of mineralized tissues. Some studies suggest that flavonoids preserve the integrity of collagen fibers and inhibit the expression of matrix metalloproteinases, which play crucial role in remodeling and degradation of the extracellular matrix. Inhibition of the proteolytic activity acts as a natural stabilizer of collagen and reduces its degradation (Ortiz et al. 2022).

## Favorable effect of quercetin on DBD

Considering strictly DBD, of all the flavonoids mentioned in this text, only the impact of quercetin was investigated. Baş and Albeniz (2022) found that quercetin (50 mg/kg for 28 days) significantly increased trabecular thickness (Tb.Th) and decreased structure model index (SMI) in diabetic rats. According to Kanter et al. (2007), trabecular bone volume (Bv/Tv), Tb.Th, trabecular number (Tb.N) were elevated after quercetin supplementation (15 mg/kg for 4 weeks) in STZ-induced diabetic rats. Improved biomechanical strength was also observed in the quercetin-treated group. Similarly, Liang et al. (2011) reported higher values of Bv/Tv, Tb.Th, Tb.N, bone mineral density (BMD), cortical bone thickness (Cr.Th), cortical bone area (C.Ar), and connectivity density (Conn.D) in diabetic rats supplemented with quercetin (30 mg/kg and 50 mg/kg during 8 weeks). On the other hand, quercetin significantly reduced SMI and trabecular separation (Tb.Sp). Histomorphometric analysis showed that both decreased bone formation and bone resorption were partially restored by quercetin. Hassan et al. (2018) examined the effect of quercetin on several bone mineralization biomarkers in diabetic patients. According to their results, quercetin (500 mg daily for 3 months) increased plasma calcium levels, 25(OH) vitamin D, and modulated bone mineralization represented by an increase in osteocalcin. Table 3 summarizes detailed characteristics and main findings of included studies.

**Table 3.** Favorable effect of quercetin on diabetic bone disease.

Complication	Applied treatment and metabolite description	Research model	Main outcomes	Reference
**DBD**	Quercetin (50 mg/kg for 28 days) Manufacturer and/or supplier of the product: Sigma-Aldrich (St. Louis, MO, USA) Product name: Quercetin Minimal active dose: 50 mg/kg	*In vivo*: male Sprague Dawley rats with STZ- and nicotinamide-induced DM (n = 10 per group) Control: positive and negative (n = 10 per group)	↑ Tb.Th ↓ SMI ↓ Blood glucose ↑ Serum GSH levels	Baş and Albeniz (2022)
	Quercetin (15 mg/kg i.p. for 4 weeks) Manufacturer and/or supplier of the product: Sigma-Aldrich (St. Louis, MO, USA) Product name: Quercetin Minimal active dose: 15 mg/kg	*In vivo*: male Wistar rats with STZ-induced DM - single i.p. injection of STZ (50 mg/kg) (n = 10 per group) Control: positive and negative (n = 10 per group)	↑ BV/TV, Tb.Th, Tb.N ↑ Biomechanical strength ↓ Blood glucose	Kanter et al. (2007)
	Quercetin (5, 30 and 50 mg/kg for 8 weeks) Manufacturer and/or supplier of the product: Sigma, Chemical Co. (St Louis, MO, USA) Product name: Quercetin Minimal active dose: 30 mg/kg	*In vivo*: male Sprague Dawley rats with STZ-induced DM - single i.p. injection of STZ (100 mg/kg) (n = 30 per group) Control: positive and negative (n = 10 per group)	↑ BMD, BV/TV, Tb.Th, Tb.N, Cr.Th, Ct.Ar, Conn.D ↓ SMI, Tb.Sp ↑ Biomechanical indices of femurs ↑ BFR/BS, MS/BS, MAR, Oc.S/BS ↓ Blood glucose ↑ Serum OC, ALP and urinary DPD/Cr ratio	Liang et al. (2011)
	Quercetin (500 mg daily for 3 months) Manufacturer and/or supplier of the product: Jarrow Formulas, Quercetin, 500 mg; product code JRW-14052 Minimal active dose: 500 mg	Clinical study: T2DM patients (n = 20, age 46.9 ± 1.77 years; 12 males, 8 females) Control group (n = 20, age 47.2 ± 1.96 years; 11 females, 9 males)	↑ Plasma calcium levels ↑ 25(OH) vitamin D ↑ Osteocalcin (compared to a pretreatment state)	Hassan et al. (2018)

Abbreviations: ↑ increase, ↓ decrease, ALP, alkaline phosphatase; BFR/BS, bone formation rate per bone surface; BMD, bone mineral density; BV/TV, relative bone volume; C.Ar, cortical bone area; Conn.D, connectivity density; Cr.Th, cortical thickness; DPD/Cr, deoxypyridinoline/creatinine; i.p., intraperitoneal; MAR, mineral apposition rate; MS/BS, mineralizing surface per bone surface; OC, osteocalcin; Oc.S/BS, osteoclast surface per bone surface; SMI, structure model index; STZ, streptozotocin; T2DM, type 2 diabetes mellitus; Tb.N, trabecular number; Tb.Sp, trabecular separation; Tb.Th, trabecular thickness.

In general, synergistic effects have been reported for multiple flavonoids. Regarding SB extracts from leaves and berries, kaempferol-3-rutinoside was the most abundant in leaves and kaempferol aglycone in berries. In contrast to berry extracts, leaf extracts contained flavan-3-ols with epicatechin in the highest concentration (Čulina et al. 2024). According to Teleszko et al. (2015), flavonoids constituted the main polyphenolic group identified in SB berries with the structures of isorhamnetin (6 compounds), quercetin (4 compounds), and kaempferol (1 compound) glycosides. The simultaneous application of kaempferol and (-)-epicatechin demonstrated antibacterial properties (Escandón et al. 2016). Combinations of kaempferol and epigallocatechin gallate exhibited synergistic antioxidant activity in HepG2 cells by scavenging ROS and up-regulating higher cellular antioxidant enzyme activities (Zhang et al. 2023). Ackland et al. (2005) revealed a synergistic response between kaempferol and quercetin in inhibiting cell proliferation in human cancer cell lines. Moreover, the berry constituents quercetin, kaempferol, and pterostilbene synergistically attenuated ROS through the involvement of the Nrf2-ARE signaling pathway (Saw et al. 2014). Additionally, herbs rich in quercetin and kaempferol have been shown to reduce symptoms of Alzheimer’s disease, which is considered a risk factor for osteoporosis (Chen and Lo 2017; Alexander et al. 2023). Furthermore, IR in the brain, known as type III diabetes mellitus (T3DM), has been observed in Alzheimer’s disease patients, which has great potential to affect neurocognition and contribute to the etiology of this disease (Nguyen et al. 2020). Therefore, the information mentioned above supports our hypothesis that SB rich in kaempferol, quercetin, isorhamnetin, and epicatechin could positively influence DBD. This assumption can be further supported by several studies (Ganju et al. 2005; Jayashankar et al. 2012; Park et al. 2022; Yuan et al. 2022) demonstrating the significant potential of SB in the treatment of other bone-related diseases such as osteoporosis and rheumatoid arthritis (Table 4).

**Table 4.** The impact of sea buckthorn on other bone-related diseases.

Disease	Applied treatment and metabolite description	Research model	Main outcomes	Reference
**Osteoporosis**	sea buckthorn (10 mL/kg twice daily for 6 weeks) Origin: purchased from a University center Minimal active dose: 10 mL/kg No data about herbal parts used, authentication of the plant material, locality and date of harvesting, deposition of voucher specimen, details about plant material processing.	*In vivo*: female OVX Sprague-Dawley rats (n = 6 per group) Control: positive and negative (n = 4 and 6 per group)	↑ Estrogen, P1NP, CTX ↑ BMD, BMC ↑ BV/TV, Tb.Th ↓ Tb.N, Tb.Sp	Yuan et al. (2022)
	sea buckthorn fruits extracts and their fractions (50 and 150 mg/kg for 12 weeks) Herbal parts: freeze-dried fruits Origin: purchased from Fuyang Bestop Import and Export, Ltd. (Fuyang City, Anhui, China) Minimal active dose: 50 mg/kg Authentication: Ki Hyun Kim, Sungkyunkwan University, Korea Voucher specimen deposited: in an institutional herbarium Extraction details: solvent (70% ethanol, distilled water, hexane, chloroform, ethyl acetate, and n-butanol), type (liquid), fractionated by preparative HPLC No data about locality and date of harvesting, extract characterization.	*In vivo*: female OVX ICR mice (n = 10 per group) Control: positive and negative (n = 6 and 10 per group)	↑ BMD ↓ Bone marrow fat ↓ Cartilage damage and disruption of trabecular bone structure ↑ ALP, OPN, RUNX2, OSX	Park et al. (2022)
**Rheumatoid arthritis**	sea buckthorn extract (200 mg/kg i.p. for 28 days) Herbal parts: fruit leaves Locality of harvesting: hilly regions of Western Himalayas, India Extraction details: solvent (70% ethanol), type (liquid) Minimal active dose: 200 mg/kg No data about authentication of the plant material, date of harvesting, deposition of voucher specimen, extract characterization.	*In vivo*: Sprague Dawley rats with adjuvant-induced arthritis (n = 8 per group) Control: positive and negative (n = 8 per group)	↓ Edema ↓ Lymphocyte proliferation (7th and 14th day) ↓ Cartilage and bone erosion, synovial hyperplasia	Ganju et al. (2005)
	sea buckhorn (270 μg/kg for 18 days) Herbal parts: leaves Extraction details: solvent (ethanol), type (liquid, supercritical CO₂ technology), drug to extract ratio 100:1.77 Minimal active dose: 270 μg/mL Methods for material characterization: HPLC No data about origin, authentication, voucher specimen deposit, locality and date of harvesting.	*In vivo*: BALB/c mice with adjuvant-induced arthritis − 100 μL of complete Freund’s adjuvant intradermally into the foot pad (n = 4 per group) Control: positive and negative (n = 4 per group)	↓ Inflammation of paw edema	Jayashankar et al. (2012)

Abbreviations: ↑ increase, ↓ decrease, ALP, alkaline phosphatase; BMC, bone mineral content; BMD, bone mineral density; BV/TV, relative bone volume; CTX, C-terminal telopeptide of type I collagen; HPLC, high-performance liquid chromatography; OPN, osteopontin; OSX, osterix; OVX, ovariectomized; P1NP, N-terminal propeptide of type I procollagen; RUNX2, runt-related transcription factor 2; Tb.N, trabecular number; Tb.Sp, trabecular separation; Tb.Th, trabecular thickness.

## Limitations of included studies

*In vitro* models using cells (e.g., HepG2, THP-1, HMC-1) and *in vivo* models using animals (mostly diabetic mice and rats) were used in this review. Although the creation of traditional 2D models is simple and affordable, certain problems have been reported such as alterations in gene expression and cell activity during the experiment (Mohandas et al. 2023). Indeed, 2D culture is unable to express the microenvironment of *in vivo* biology that affects cell physiology. Previous studies have shown that the extracellular matrix is essential for cellular activity and that 3D cell culture can more accurately simulate the *in vivo* microenvironment than 2D culture systems (Yudhani et al. 2023). To demonstrate spontaneous T1DM, genetically engineered rodents were used. Similarly, T2DM has been studied in rodents by mimicking metabolic dysregulation, IR, and β-cell dysfunction using chemical, surgical, and genetic interventions.

The underlying physiological, genetic and molecular mechanisms, however, clearly differ between humans and other animals due to species-specific differences. Despite similarities in DM phenotype, the architecture of islets of Langerhans has been demonstrated to differ between humans and rodents (Steiner et al. 2010). In addition, STZ is the most important diabetogenic chemical widely used in experimental animals to establish animal models of T1DM and/or T2DM. Both a single high dose of STZ and multiple injections of a low dose of STZ over 5 consecutive days have been used to establish T1DM in rats. In animal models of T2DM, STZ injection can be administered after high-fat diet induction, nicotinamide injection, or during the neonatal period (Srinivasan and Ramarao 2007; Ghasemi et al. 2014). In general, males are known to be more vulnerable to STZ than females, and while some strains of rats (e.g., Wistar and Sprague-Dawley rats) are sensitive to STZ, others (e.g., Wistar-Kyoto rats) are less sensitive. Although intravenous injection of STZ results in more stable hyperglycemia, it is usually administered intraperitoneally (Ghasemi and Jeddi 2023). Thus, it is recommended to provide details in the method section such as animal age, sex, preparation of STZ to avoid poor concordance between preclinical and clinical studies. It is therefore evident that the findings of animal model studies can be used to understand disease mechanisms and test hypotheses to be verified in clinical studies (Wall and Shani 2008; Gao et al. 2024). Although rodent models are widely used in biomedical research due to their low cost and clear genetic basis, translational research often requires large animal models to simulate the human disease state as closely as possible (Hu et al. 2022). In the future, more multidisciplinary translational research should be conducted leading to the potential use of obtained results in the clinical setting (Wang et al. 2021).

Another limitation is the lack of information on the plant material used, especially in studies investigating SB extracts, as recommended by the best practice guidelines (Heinrich et al. 2020, 2022), suggesting lower reliability of these studies. Furthermore, the known interference of flavonoids with commonly used assays (such as MTT or protein assays) should always be considered (Singh et al. 2020). These characteristics of flavonoids might cause misleading findings in molecular docking analysis, so experimental research should confirm them (Martiniakova et al. 2024b). The low oral bioavailability of flavonoids due to their poor water solubility could be another disadvantage. Absorption enhancers, structural transformation, and pharmaceutical technologies are some of the promising approaches that have been developed and used to address this issue. These strategies can effectively improve the oral bioavailability of flavonoids by elevating their solubility, dissolution rate, and permeability; preventing their metabolism in the gastrointestinal tract; and/or delivering them directly to their physiological targets (Zhao et al. 2019).

Although many studies have confirmed the safety of flavonoids, in higher doses they can be toxic, as they can act as mutagens, pro-oxidants that generate ROS, and as inhibitors of significant enzymes involved in hormone metabolism (Skibola and Smith 2000). In general, excessive intake of flavonoids is associated with carcinogenicity and mutation, kidney and liver damage, effects on reproductive and thyroid function, and disturbances in intestinal flora. Available data suggest that natural flavonoid glycosides act on multiple targets at different doses in both *in vitro* and *in vivo* studies, suggesting a quite complicated mechanism of toxicity (Tang and Zhang 2022; Mehjabin et al. 2024). Although no tolerable upper dietary reference intake has been established for flavonoids, the use of supplements in commonly recommended gram rather than milligram doses could lead to exposure to potentially toxic levels. For example, typical recommended doses of quercetin from manufacturers range between 500 mg and 1 g per day, which is 10 to 20 times more than would be consumed in a typical vegetarian diet (Skibola and Smith 2000). Therefore, avoiding high doses of supplementation (and possibly monitoring side effects) is essential to prevent potential toxicity. Furthermore, other factors can also affect bioavailability and biological activity of flavonoids, including enzymes involved in their biotransformation, gut microbiota, age, gender, race, and metabolic diseases (Hu et al. 2025). Genetic polymorphisms in genes encoding biotransformation enzymes may cause individual differences in bioavailability. Different composition and activity of the gut microbiota may lead to alterations in the absorption, distribution, metabolism, and excretion of flavonoids (Favari et al. 2024). In addition, dietary composition influences the bioavailability of flavonoids; for example, a high-fat diet may increase the absorption of lipophilic flavonoids such as quercetin (Makino et al. 2018). On the other hand, recent data suggest that by modifying flavonoid molecules, it is possible to prepare compounds with lower toxicity, which could contribute to solving the toxicity problem (Vučkovski et al. 2024).

## Conclusions and perspective

This review offers, for the first time, a comprehensive description of the positive relationships between SB, its most abundant flavonoids, and key mechanisms associated with DBD. It also demonstrates the favorable impact of quercetin on DBD, as the relationships between SB, isorhamnetin, kaempferol, and DBD has not been investigated yet. Considering that SB has already shown significant promise in the treatment of osteoporosis and rheumatoid arthritis, and synergistic effects between quercetin and kaempferol, kaempferol and epicatechin have also been revealed, its promising potential to alleviate DBD is anticipated. The limited bioavailability of flavonoids makes clinical application difficult, although they have demonstrated encouraging impacts in many studies. Nevertheless, developments in drug delivery technologies represent viable ways to improve their therapeutic efficacy. Other limitations of several studies presented in this review include insufficient information on the plant material obtained, the animal model used, small sample size, model-specific constraints, and generalizability to human subjects. For the reasons mentioned above, further high-quality *in vitro* and animal model studies, as well as large-scale, multicenter, and prospective clinical trials involving both diabetic and non-diabetic patients, are needed to confirm the potential beneficial impact of SB on DBD and to find more effective therapies for bone-related complications associated with DM.

## Data Availability

The authors confirm that the data supporting the findings of this study are available within the article and its supplementary materials.
